# What is the trigger mechanism for the reversal of electron flow in oxygen-tolerant [NiFe] hydrogenases?†
†Electronic supplementary information (ESI) available: The supplementary information contains computational details, representation of models used, additional figures, pictures and coordinates for the calculated structures in Fig. 6, and pictures and coordinates for some other relevant calculated structures. See DOI: 10.1039/c4sc03223c



**DOI:** 10.1039/c4sc03223c

**Published:** 2014-12-08

**Authors:** Ian Dance

**Affiliations:** a School of Chemistry , University of New South Wales , Sydney 2052 , Australia . Email: i.dance@unsw.edu.au

## Abstract

The [NiFe] hydrogenases use an electron transfer relay of three FeS clusters – proximal, medial and distal – to release the electrons from the principal reaction, H_2_ → 2H^+^ + 2e^–^, that occurs at the Ni–Fe catalytic site. This site is normally inactivated by O_2_, but the subclass of O_2_-tolerant [NiFe] hydrogenases are able to counter this inactivation through the agency of an unusual and unprecedented proximal cluster, with composition [Fe_4_S_3_(S^cys^)_6_], that is able to transfer two electrons back to the Ni–Fe site and effect crucial reduction of O_2_-derived species and thereby reactivate the Ni–Fe site. This proximal cluster gates both the direction and the number of electrons flowing through it, and can reverse the normal flow during O_2_ attack. The unusual structures and redox potentials of the proximal cluster are known: a structural change in the proximal cluster causes changes in its electron-transfer potentials. Using protein structure analysis and density functional simulations, this paper identifies a closed protonic system comprising the proximal cluster, some contiguous residues, and a proton reservoir, and proposes that it is activated by O_2_-induced conformational change at the Ni–Fe site. This change is linked to a key histidine residue which then causes protonation of the proximal cluster, and migration of this proton to a key μ_3_-S atom. The resulting SH group causes the required structural change at the proximal cluster, modifying its redox potentials, and leads to the reversed electron flow back to the Ni–Fe site. This cycle is reversible, and the protons involved are independent of those used or produced in reactions at the active site. Existing experimental support for this model is cited, and new testing experiments are suggested.

## Abstract 2

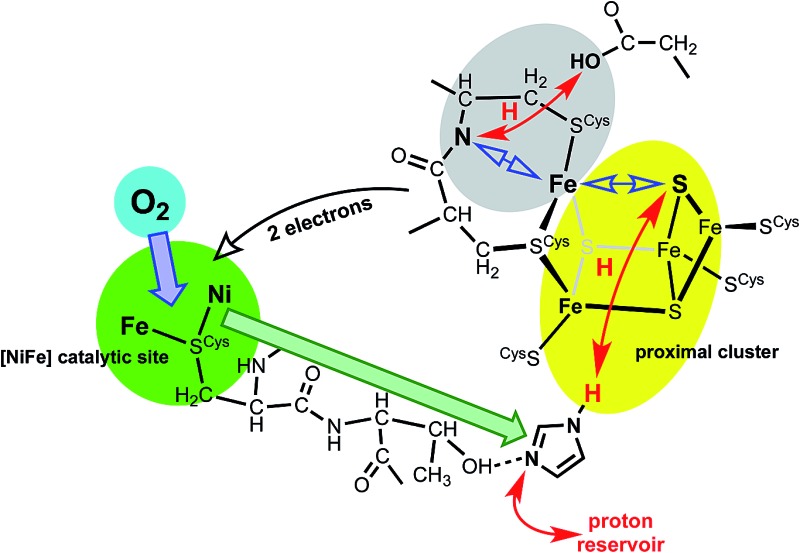
A new mechanistic model is developed for the sequence of events by which oxygen-tolerant [NiFe] hydrogenase enzymes respond to O_2_.

## Introduction

The hydrogenase enzymes catalyse the reactions H_2_ ↔ 2H^+^ + 2e^–^, and occur in two main groups, those with an [FeFe] active site and those with an [NiFe] active site.^1–4^ The [FeFe] enzymes mainly reduce protons, while the [NiFe] enzymes mainly oxidise H_2_. These hydrogenases are generally very sensitive to O_2_, but there is a group of [NiFe] hydrogenases found in aerobic bacteria that are able to oxidise H_2_ in the presence of O_2_.^5–10^ The ability of these O_2_-tolerant hydrogenases to concurrently oxidise H_2_ and reduce O_2_ is of obvious chemical and technological interest.^7,9,11–16^

**Scheme 1:**
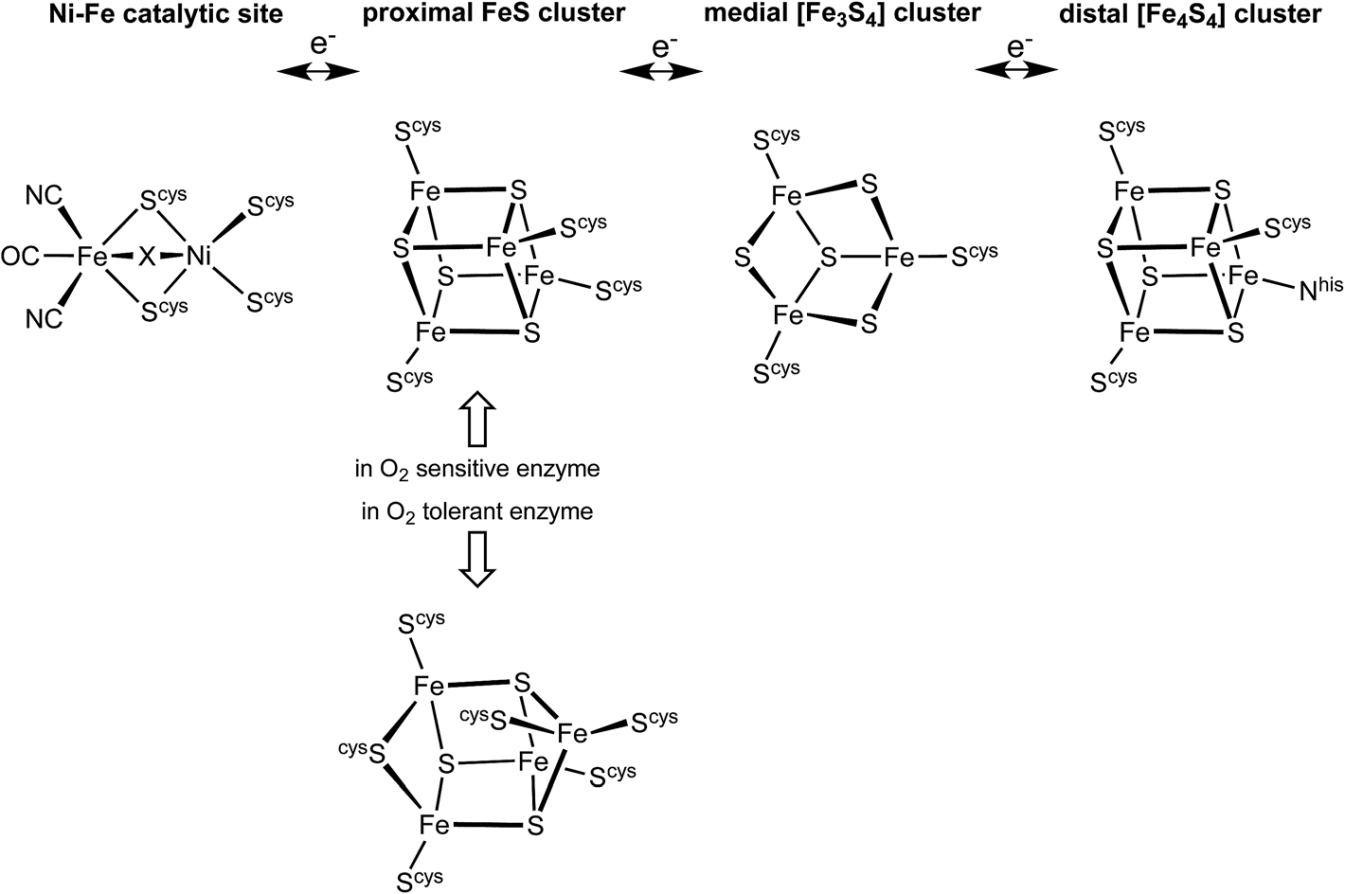
The electron transfer relay of metal clusters in [NiFe] hydrogenases, with the different proximal clusters related to O_2_ sensitivity. At the Ni–Fe catalytic site X is normally H or absent, but is OH or O_2_H in inactive states.

There is substantial knowledge of the protein structures and of the intermediates and mechanism for normal reactivity at the (^cys^S)_2_Ni(μ-S^cys^)_2_Fe(CN)_2_(CO) catalytic site (Scheme 1).^2,3,9,17–23^ The inactivation caused by O_2_ introduces OH or O_2_H species at the bridging X site, forming inactive species that are ‘ready’ (Ni-B) or ‘unready’ (Ni-A) for reactivation.^1,8,19,24–26^ A key attribute of the O_2_-tolerant enzymes is modification of the redox potentials in the chain of three FeS clusters (Scheme 1) that transfer electrons to and from the catalytic site.^5,7,8^ In recent years the crystal structures of these O_2_-tolerant enzymes from species *Hydrogenovibrio marinus* (*Hm*), *Ralstonia eutropha* (*Re*), *Escherichia coli* Hyd-1 (*Ec*Hyd-1) and *Salmonella enterica* Hyd-5 (*Se*Hyd-5) have revealed their distinctive structural property, which is the presence of an unusual and unprecedented Fe_4_S_3_(S^cys^)_6_ cluster^27–31^ in place of the standard Fe_4_S_4_(S^cys^)_4_ cluster at the proximal location, adjacent to the [Ni–Fe] active site, in the electron transfer chain.

This proximal cluster occurs in three oxidation states, described in terms of the core charges as [Fe_4_S_3_]^3+^ reduced (RED), [Fe_4_S_3_]^4+^ oxidised (OX), and [Fe_4_S_3_]^5+^ super-oxidised (SOX). The reduction potentials for the one-electron steps between these states are anomalously close, namely +232 mV (SOX/OX) and +87 mV (OX/RED) in *Aquifex aeolicus*,^32^ (and *ca.* +160 mV, –60 mV in *Alcaligenes eutrophus* ^6,33^). In *Hm* the two successive potentials are +230 and +30 mV, and in a slightly modified form of *Hm* are +175 and +90 mV.^34^ This close separation of *ca.* 150–200 mV between the [Fe_4_S_3_]^5+/4+^ and [Fe_4_S_3_]^4+/3+^ potentials contrasts the normal separations between successive one-electron transfer potentials for [Fe_4_S_4_] clusters of 600 to 1000 mV,^35–37^ and suggests that a structural change occurs in the sequence RED–OX–SOX.

**Fig. 1:**
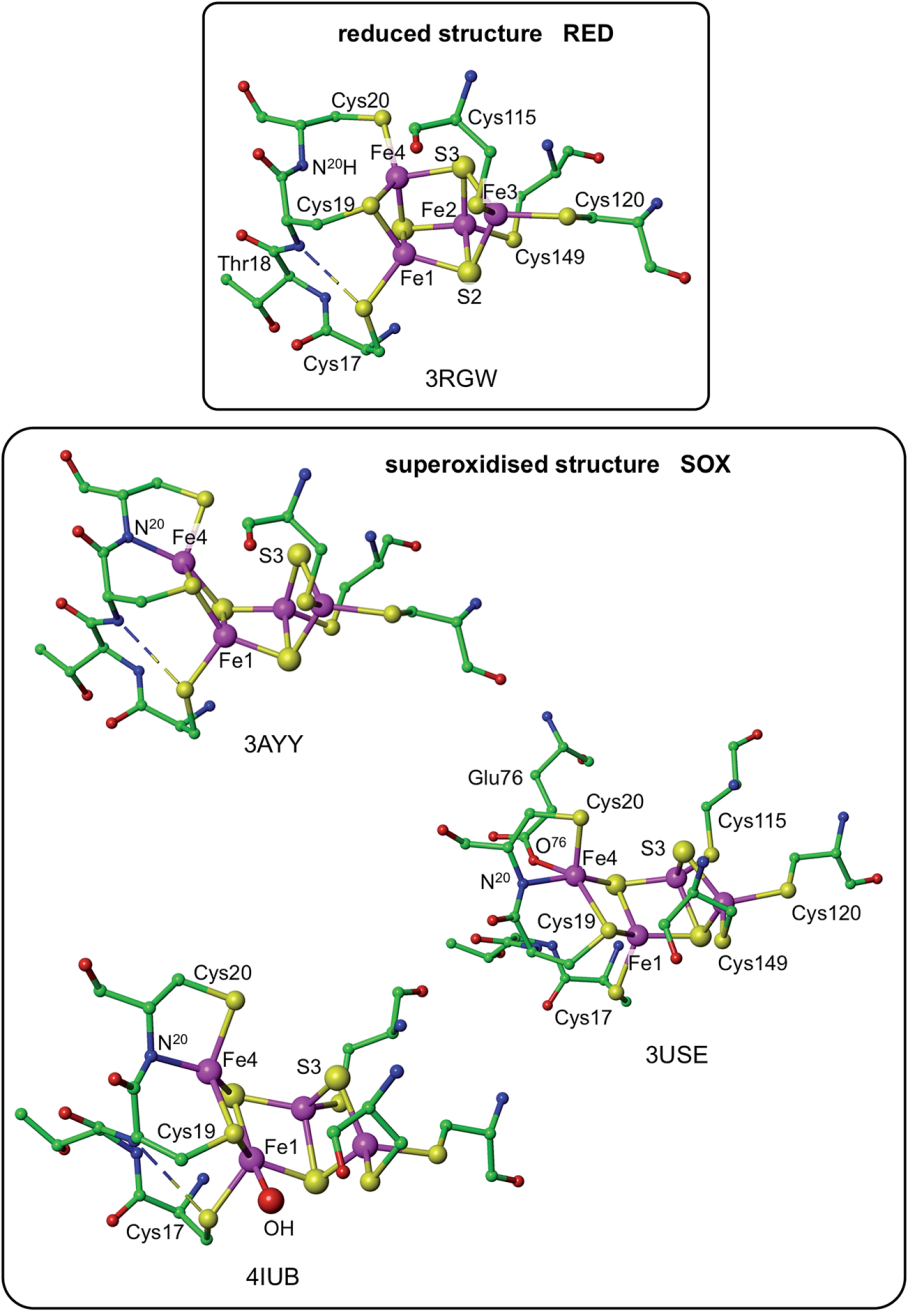
Structures of the reduced (RED, upper panel) and superoxidised (SOX, lower panel) states of the proximal cluster. PDB codes are marked [3RGW*Ralstonia eutropha* (*Re*),^28^; 3AYY*Hydrogenovibrio marinus*,^27^; 3USE*Escherichia coli* Hyd-1 (*Ec*Hyd-1),^29^; 4IUB*Ralstonia eutropha*,^30^] and atom/residue numbering is that of the *Re* and *Ec*Hyd-1 structures. In the reduced cluster ; 3RGW the Fe4–S3 and Fe4–N^20^ distances are bonding (2.31 Å) and non-bonding (3.29 Å), while in the superoxidised form these interactions are reversed, Fe4–S3 4.01, 3.90 Å, Fe4–N^20^ 2.09, 2.11 Å. The SOX form has been observed with an OH ligand on Fe1 (; 4IUB), and a bonding interaction between the side-chain of Glu76 and Fe4 was detected for *Ec*Hyd-1 (; 3USE). The broken line is a hydrogen bond from N^18^–H to S^17^.

Three crystallographic investigations^27–29^ have revealed that the structure of the RED form of the proximal cluster in three different species is that shown in the upper panel of Fig. 1. The RED cluster is derived from standard cubanoid Fe_4_S_4_(SR)_4_ by the opening of one Fe–(μ_3_-S) bond, replacement of μ_3_-S with doubly-bridging Cys19 thiolate, and adding another cysteine to the released Fe3 atom (the atom numbering used here is that of *Re* ^28^ and *Ec*Hyd-1^29^), resulting in the composition Fe_4_(μ_3_-S)_3_(μ-SR)(SR)_5_. The super-oxidised SOX structure of the proximal cluster is distinctly different (Fig. 1, lower panel): the Fe4–S3 bond no longer exists, and the main chain amide of Cys20 is deprotonated and rotated so as form a bond to Fe4, maintaining the four-coordination of Fe4. This core structure is even more open than that of RED. Variations in the structure of the SOX form have been reported in the diffraction analyses of crystals prepared in different ways, and from different organisms. Crystals from *Ec*Hyd-1 showed evidence of variable conformations of the Glu76 side-chain, and of its coordination to Fe4 (Fig. 1, ; 3USE).^29^ A recent crystallographic analysis of the SOX form of *Re* (PDB ; 4IUB, Fig. 1)^30^ revealed an additional OH ligand at Fe1. Further evidence for the OH ligand on Fe1 in the *Re* crystals has been provided, together with possible reasons for non-detection of this OH in the *Hm* and *Ec*Hyd-1 crystals.^30^ On the basis of the controlled redox state of their crystals, Frielingsdorf *et al.* ^30^ concluded that the structure of the proximal cluster at the intermediate OX level is very similar to that of RED. The structures of the proximal cluster in the RED and SOX states are unprecedented.

The structures of the three redox states of the proximal cluster reveal the reason for their unusual reduction potentials. In the SOX form the replacement of a μ_3_-S ligand on Fe4 by deprotonated amide stabilises oxidised Fe^38^ and markedly lowers its reduction potential to a value in the physiological potential range and only *ca.* 150 mV more positive than the OX reduction potential.^7,8,28,29,32,34,37^ Additional coordination of OH^–^ on Fe1 would also stabilise the oxidised cluster.^30^

**Fig. 2:**
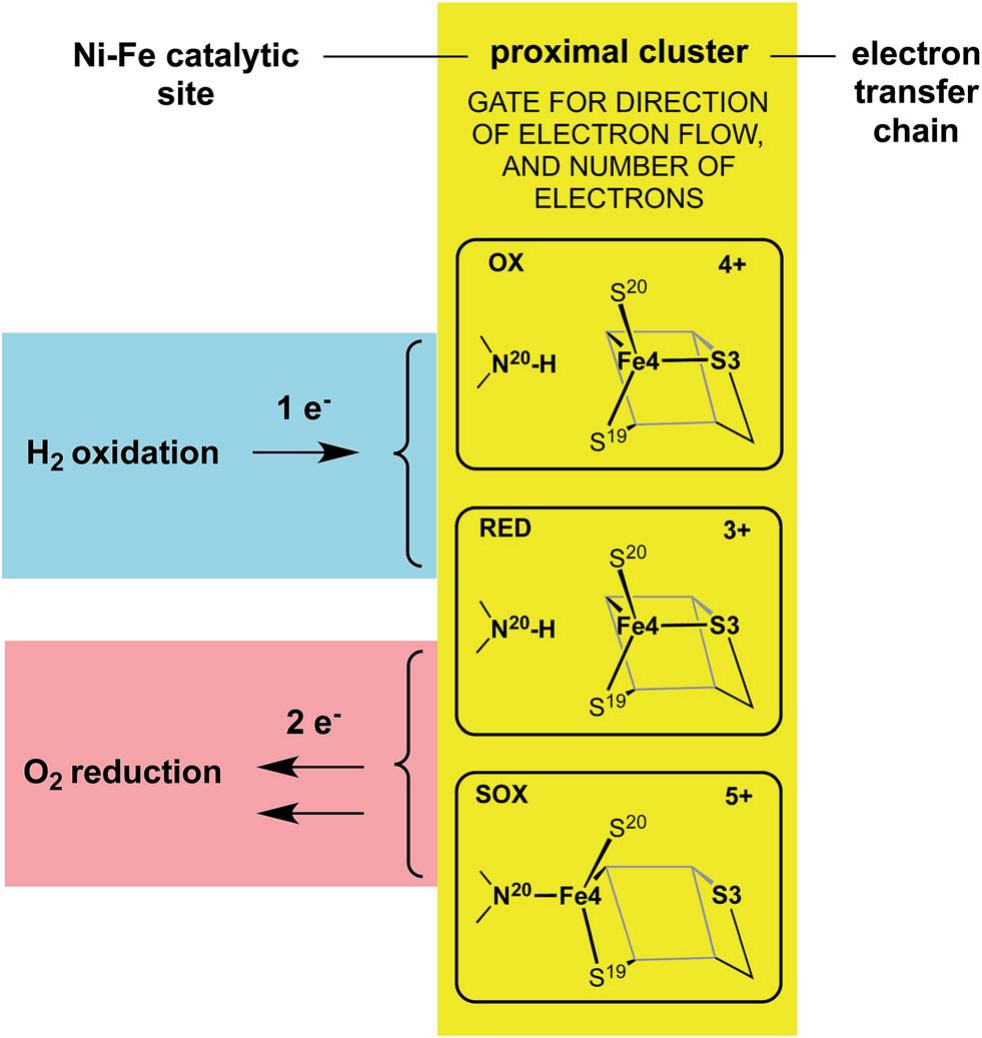
The gating function of the proximal cluster, accepting one electron during oxidation of H_2_, or providing two electrons towards reduction of O_2_.

The redox potentials of the proximal cluster allow it to collect one electron (OX → RED) from the Ni–Fe site as part of the H_2_ oxidation cycle, and to discharge two electrons (RED → SOX) to the Ni–Fe site when it is necessary to enable four-electron reduction of O_2_ (the other two electrons are believed to come from the medial cluster and the Ni–Fe site) and avoid inactivation of the Ni–Fe site.^6,8,10,24–26,30,34^ The proximal cluster has a key function in gating either *one electron from* or *two electrons to* the catalytic site (Fig. 2).

## Questions: what is the trigger mechanism?

These data and model just outlined provide a plausible account of the unusual *ability* of the electron transport chain to move electrons in two opposing directions depending on the substrate, H_2_ or O_2_, that presents at the Ni–Fe catalytic site. In the presence of O_2_ the proximal cluster adopts the SOX structure when it releases two ‘rescue’ electrons.^34^ How can the catalytic site, when challenged by O_2_, signal to the proximal site the need for two electrons, and cause their delivery? The key must be the geometrical change that occurs at the proximal cluster and changes its OX/SOX potential from an otherwise inaccessible value (at least 500 mV too positive) to a value close to the RED/OX potential. What chemical event at the proximal cluster causes the necessary geometrical changes, the severing of the Fe4–S3 bond, the deprotonation of N^20^–H, and the formation of the N^20^–Fe4 bond? This is the central question addressed in the investigation reported here.

The auxiliary question also considered here is the signaling mechanism by which the presence of O_2_ at the Ni–Fe site communicates to the proximal cluster this need for structural change.

I note in passing that the known correlations between geometrical structure and redox potential at the proximal cluster are based on equilibrium measurements, namely redox titrations and crystal structures. These describe thermodynamic states of the system, but the key question raised is kinetic and mechanistic. Expressed in electrochemical terms, the changes at the proximal cluster involve electron transfer (E) steps and chemical steps (C): the nature and sequence of the coupling of these is a relevant question, that could in principle be answered through kinetic electrochemical experiments such as variable scan-rate cyclic voltammetry.

## Results

**Fig. 3:**
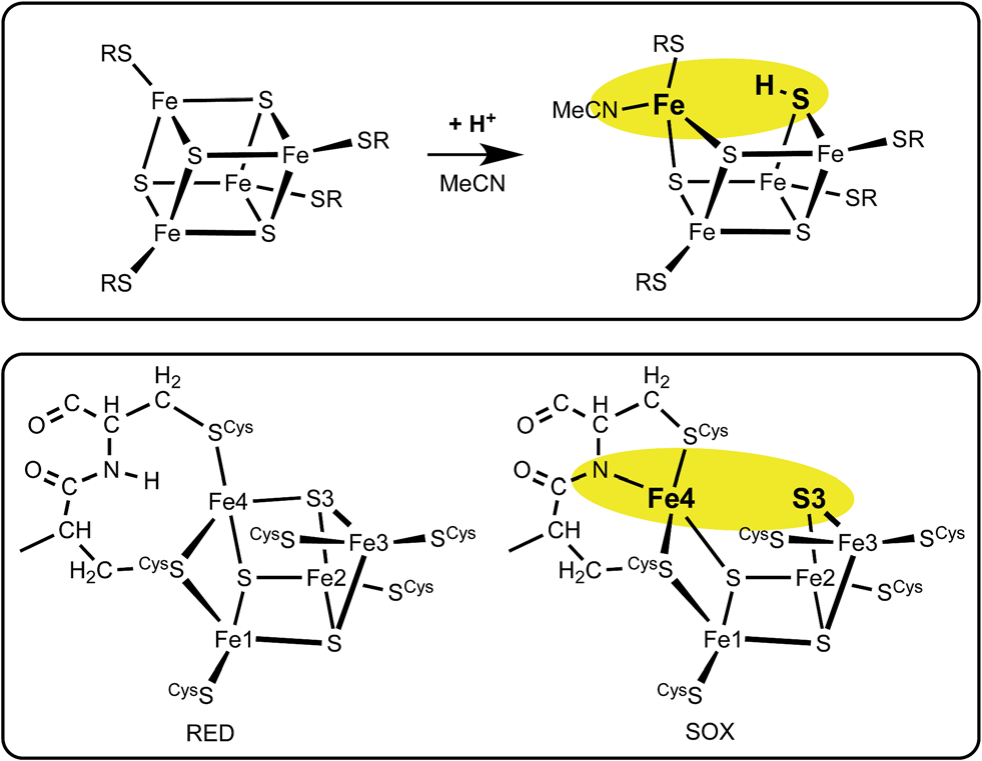
The similarity between the change on protonation of [Fe_4_S_4_(SR)_4_]^2–^ in acetonitrile (upper panel), and the structural change RED → SOX in the proximal cluster (lower panel).

A clue to the trigger for structural change comes from recent density functional simulations (inspired by investigations of proton transfer aspects of the mechanism of nitrogenase^39–41^) of the acid-catalysed substitution reactions of [Fe_4_S_4_X_4_]^2–^ clusters.^42,43^ The primary step in this catalysis is protonation of μ_3_-S, which causes one S–Fe bond to break leaving three-coordinate Fe plus μ-SH: the under-coordinated Fe is coordinated by solvent (acetonitrile). From this point the substitution mechanism develops in a rational fashion: a large amount of kinetic data has now been satisfactorily interpreted, based on the structural rearrangements that occur as a result of protonation of μ_3_-S.^43^ Fig. 3 outlines the calculated structural change on protonation of [Fe_4_S_4_(SR)_4_]^2–^, in comparison with the structural change in the proximal cluster. The obvious hypothesis is that protonation of S3 is involved.

The computational methods and models used in this work are described in the ESI.† I modeled the proximal structure type computationally, first as [Fe_4_S_3_(SMe)_6_]^3–, 2–, 1–^ encompassing the three core redox levels [Fe_4_S_3_]^3+, 4+, 5+^. In all three redox states the cluster structure closely resembles the closed geometry of the *reduced* proximal cluster. The closed structure of [Fe_4_S_3_(SMe)_6_] occurs unchanged as a local energy minimum when it is oxidised by two electrons. Using a more elaborate model, Pelmenschikov and Kaupp also recorded a local energy minimum for the closed geometry when oxidised by two-electrons.^44^ Subsequently, using even more complete models for the proximal cluster (ESI Fig. S4†), I have confirmed that the closed structure when oxidised by two electrons does not undergo barrierless opening, and further that the atom charges, spin densities, and interatomic distances change by remarkably small increments as the core [Fe_4_S_3_] is oxidised from charge +3 to +4 and +5 (ESI Tables T2 and T3).†

The key results for the [Fe_4_S_3_(SR)_6_] structure type are (1) the cluster is not opened geometrically by redox change, (2) the cluster is opened by protonation of the relevant μ_3_-S atom, with the Fe–S(H) distance increasing by *ca.* 1 Å, (3) this opening is reversed on deprotonation, and (4) this protonation behaviour is independent of the redox level of the cluster. The findings support the premise that protonation of S3 could trigger the structural change of the proximal cluster in O_2_-tolerant hydrogenases.

### The proximal cluster as a closed protonic system

**Fig. 4:**
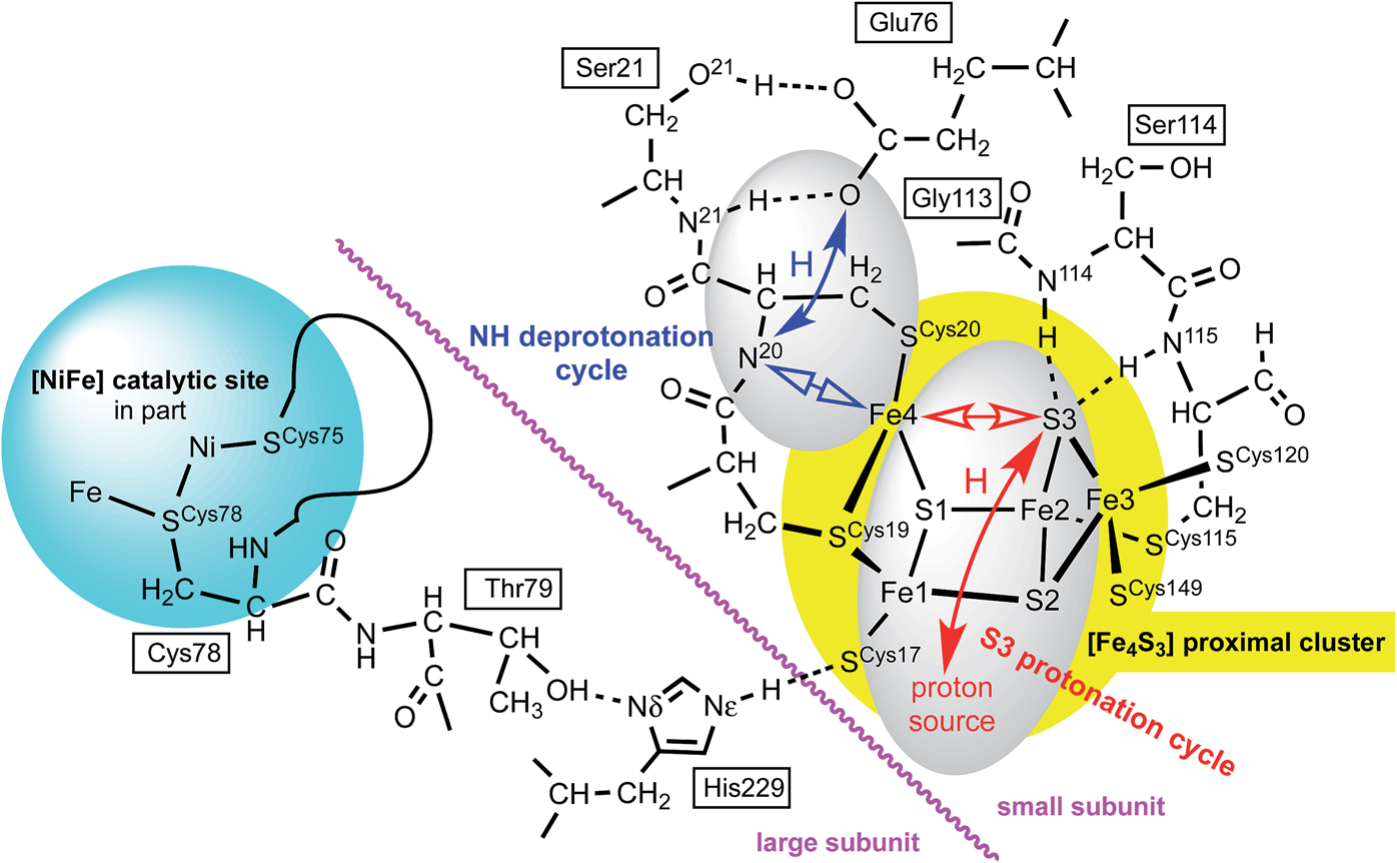
Some relevant features of the model developed in this paper. Atom numbering of *Re* and *Ec* structures is used: for *Hm* species the link residues are Cys79, Thr80, His230. Closed arrows represent proton movements; open arrows show the movement of Fe4. The separation of the NH deprotonation cycle (blue) and S3 protonation cycle (red) is evident: details of the ‘proton source’ are described in the text.

The proposed protonation of S3 to open the proximal cluster and elongate the S3–Fe4 interaction is coupled to the separate deprotonation of the peptide NH of cys20, and formation of the Fe4–N^20^ bond (Fig. 1). These two proton transfer cycles at the proximal cluster are named the S3 protonation cycle and the NH deprotonation cycle, as on Fig. 4. A plausible mechanism for the transfer of N^20^–H to the side-chain of Glu76 has been proposed, and developed with DF calculations,^29,37,44^ and is depicted in ESI Fig. S3.† It is not geometrically feasible for the proton released from N^20^–H to reach S3.

The two catalysed reactions that occur at the Ni–Fe site, oxidation of H_2_ and reduction of O_2_, involve continuous provision of protons to or from the protein surrounds. Possible pathways for these proton transfers between the Ni–Fe catalytic site and the protein surface, and between the proximal cluster and protein surface, are evident in the protein structures and have been discussed.^1,27,29,30,45^ Shomura *et al.* ^27^ and Frielingsdorf *et al.* ^30^ have suggested two different proton pathways between the Ni–Fe site and the proximal cluster. However, when the enzyme is turning over with H_2_ only, or when it is repeatedly reactivating itself under oxic conditions (*e.g.* ∼2500 turnovers h^–1^ under 10% O_2_, without degradation^26^), a continuous stream of protons is involved, and this proton stream can only connect with external solvent. The proximal cluster and the single-proton transfer pathways between it and the Ni–Fe site could not be the source or sink for the multiple protons in reactions involving H_2_ or O_2_ at the catalytic site. The mechanisms to be developed for the proton transfer steps at the proximal cluster must be readily reversible, and it is assumed that there is no change in the total number of protons at this site during each reaction cycle: the proximal cluster is postulated to be a closed protonic system.

### The linkage between the proximal cluster and Ni–Fe site

There is a direct linkage between the Ni–Fe catalytic site and the proximal cluster, illustrated in Fig. 4. One of the key cysteines bridging Ni and Fe, Cys78^L^, is flanked by Thr79^L^, which is hydrogen bonded to the Nδ atom of His229^L^ (^L^ and ^S^ denote the large and small protein subunits). In the protein crystal structures where no additional OH ligand was detected on Fe1, the Nε atom of His229^L^ is hydrogen bonded to the sulfur atom of Cys17^S^ in the proximal cluster, while in the two crystals (; 4IUB, 4IUC) containing the super-oxidised proximal cluster of *Re* the hydrogen bond from Nε of His229^L^ is directed at the OH ligand on Fe1. This link, [Ni–Fe]-Cys^L^-Thr^L^···His^L^···[proximal cluster] occurs in all three species with crystal structures, and these residues are conserved through 19 [NiFe] hydrogenases.^28^ Frielingsdorf *et al.* report mutants with His229^L^ substituted by alanine, methionine or glutamine, and conclude that His229^L^ is crucial for O_2_ tolerance.^30^ The Ni–Fe catalytic site and the proximal cluster are located in the large and small sub-units respectively, with the linkage crossing between the subunits at the His229^L^Nε hydrogen bond (Fig. 4).

Therefore it is postulated that the presence of O_2_ at the Ni–Fe site causes conformational changes that are transmitted through the Cys^L^-Thr^L^···His^L^ link to the proximal cluster. A protein conformational change caused even by the ingress of O_2_ (larger than H_2_) could also be transmitted through the interface between the large and small subunits. This mechanism for initiation of events at the proximal cluster due to changes at the Ni–Fe site does not require transfer of protons between the two cluster sites.

### Dual proton migrations in the proximal cluster

Preliminary calculations on the RED proximal cluster, using a model that included cysteines 17, 19 and 20 with normally protonated peptide N^20^–H, confirmed that *endo* protonation of S3 causes it to separate from Fe4, without changing the N^20^–H backbone, yielding three-coordinate Fe4. This means that the deprotonation of N^20^–H and formation of the N^20^–Fe4 bond are not caused by protonation of S3. The proton movements involving N^20^, already described by Pelmenschikov and Kaupp^44^ (see ESI Fig. S3†), are *spatially* separate from proton movements to and from S3.

The model developing from these considerations has two distinct proton transfer domains, and dual cycles, illustrated in Fig. 4. The protons involved in the NH deprotonation cycle (blue) and the S3 protonation cycle (red) are spatially separate and involve different protons. Each cycle involves bond breaking/making (open arrows) around Fe4, which is likely to control the *temporal* relationship between the two cycles. The NH deprotonation cycle is considered to be the same as that already described by Pelmenschikov and Kaupp,^44^ who did not consider protonation of S3 (ESI Fig. S3†). The analyses and simulations developed in this paper deal only with the S3 protonation cycle. Protonation of S3 in the protein can occur only in the *endo* conformation, in which the S3–H bond is directed towards Fe4. The alternative *exo* conformation for S3–H would also cause elongation of the S3–Fe4 bond, but the *exo* side of S3 is blocked for protonation by two well-developed peptide N–H–S3 hydrogen bonds from Cys115^S^ and Ser114^S^ (Fig. 4). Calculations on a model (ESI Fig. S1(a), also S4†) that included residues 115, 114, and 113 showed that the N^115^–H–S3 and N^114^–H–S3 hydrogen bonds do not control the position of S3 but do block its protonation on the *exo* side.

### Proton source, and the role of His229

What is the source of the proton that could reach S3? The OH group found on Fe1 in oxidised *Re* is indicative of an OH or OH_2_ ligand associated with Fe1 as a possible source. This OH/OH_2_ ligand was not detected in crystal structures of the *Hm* and *Ec* proteins, but may be present. Should this OH or OH_2_ ligand be absent from the coordination sphere of Fe1 in some forms of the proximal cluster, there is indication that it could be readily formed, because all crystal structures contain a conserved water molecule located about 4.5 Å from Fe1, and there are no intervening atoms to interfere with its movement to and ligation of Fe1 (ESI Fig. S6†). This water molecule could be involved in an associative-dissociative equilibrium with Fe1, and could be a source of the proton that can move, reversibly, to S3. However, as described below, it is not necessary to use water as the proton source.

**Fig. 5:**
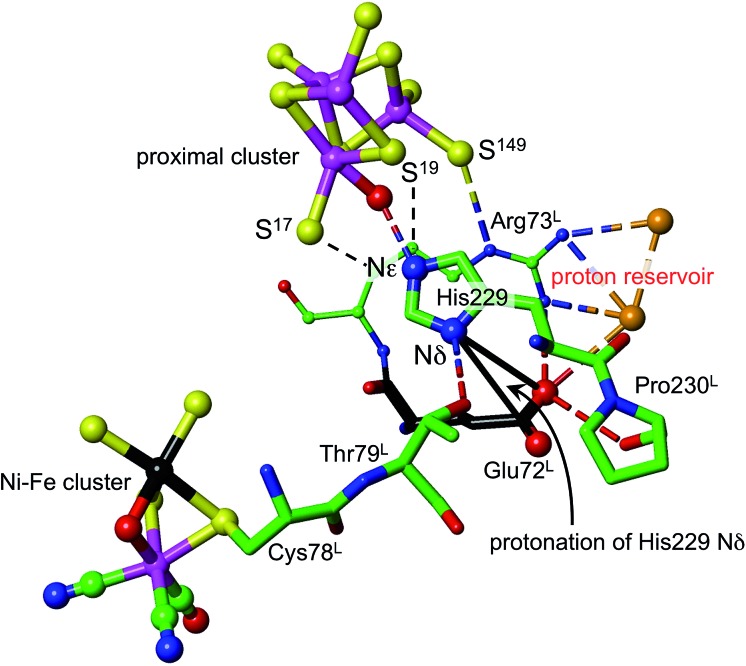
The surroundings of His229 in PDB 4IUB (which contains OH on Fe1 of the proximal cluster, and bridging O in the Ni–Fe site, the Ni–B state). Nε of His229 is hydrogen bonded to OH on Fe1, and is within hydrogen bonding distances of S^17^ (3.3 Å) and S^19^ (3.7 Å). Nδ of His229 is hydrogen bonded to the side-chain of Thr79^L^, and is near (4.2 Å, black connectors) the carboxylate side-chain atoms (red spheres) of Glu72^L^. These same carboxylate atoms are engaged in a hydrogen bonding network involving two water molecules (orange spheres), the side-chain NH_2_ groups of Arg73^L^, and carbonyl of Pro230^L^. This is the proton reservoir domain. Additional hydrogen bonds between the proton reservoir and its surrounds are not shown.

Density functional simulations of OH_2_ bound to Fe1 as a source of the proton to move towards S3 confirmed an expectation that as such it would be insufficiently acidic. The conjugate Fe–OH group is too basic to release a proton to the sulfide and cysteinyl sulfur atoms as would be required for protonation of S3. Nonligated water is also insufficiently acidic to protonate the proximal cluster. However, His229 that is able to hydrogen bond to this OH_2_/OH entity is capable of relieving the conjugate basicity of OH by provision of a proton from its Nε, particularly when pushed to do so by protonation of His Nδ. Examination of the protein structure reveals how His Nδ can be protonated. Fig. 5 depicts the relevant surroundings of His229^L^ in structure PDB ; 4IUB, the oxidised form with OH on Fe1 and bridging O in the Ni–Fe site. The other crystal structures have the same or very similar surrounds (His229 Nε is hydrogen bonded to S^17^ when OH is absent). The significant structural property is the placement of the carboxylate side-chain of Glu72^L^ close to Nδ of His229 and readily able to transfer a proton to it with minor side-chain movements. This Glu carboxylate side-chain is hydrogen bonded to two water molecules (three in PDB ; 3UQY) and to the NH_2_ side-chain groups of the adjacent residue Arg73^L^. The (H_2_O)_2_ + (–NH_2_) + (–NH_2_^+^) domain so constituted can readily function as proton reservoir, and the carboxylate side-chain can easily relay a proton to or from Nδ of His229. These features of the His229 surrounds occur in the crystal structures of all of the O_2_-tolerant proteins. Therefore it is concluded that His229 can be readily protonated, and thereby is able to function as promoter of the acidity required to push a proton onto atoms of the proximal cluster and to S3.

As shown in Fig. 5, Nε of His229 can hydrogen bond to the OH ligand if present, or to S^17^ (as observed in all other reduced and oxidised crystal structures), or, with small movement of the His229 side-chain, to S^19^. Further, density functional modeling shows that a water molecule can be hydrogen bonded in various geometrically favourable ways in the space between S^19^, S^149^, Nδ of His229, with or without bonding to Fe1. This propitious geometry allows a number of mechanistic hypotheses for transfer of a proton onto S^19^ or S^149^ of the proximal cluster, preparatory to continuing transfer to S3. The steps involved in these possibilities for preparatory protonation have been investigated using density functional simulation of the trajectories and transition states involved. This exploration revealed interesting relevant chemistry of the protonated cluster, including (a) aspects of the relative basicities of S^149^ (non-bridging thiolate), S^19^ (bridging thiolate), OH_2_ and Nε, (b) weakened ligation by protonated cysteine, (c) the ability of bridging Cys19 to reversibly unbridge from Fe4 or Fe1, and (d) the geometrical flexibility of the cysteine side-chains. These explorations of possibilities also pointed to a mechanism involving direct protonation of S^19^ by His229, without participation of any water present. This favourable mechanism is described in the following section: some additional results are provided in the ESI material.†

### Proposed mechanism for protonation of S3

**Fig. 6:**
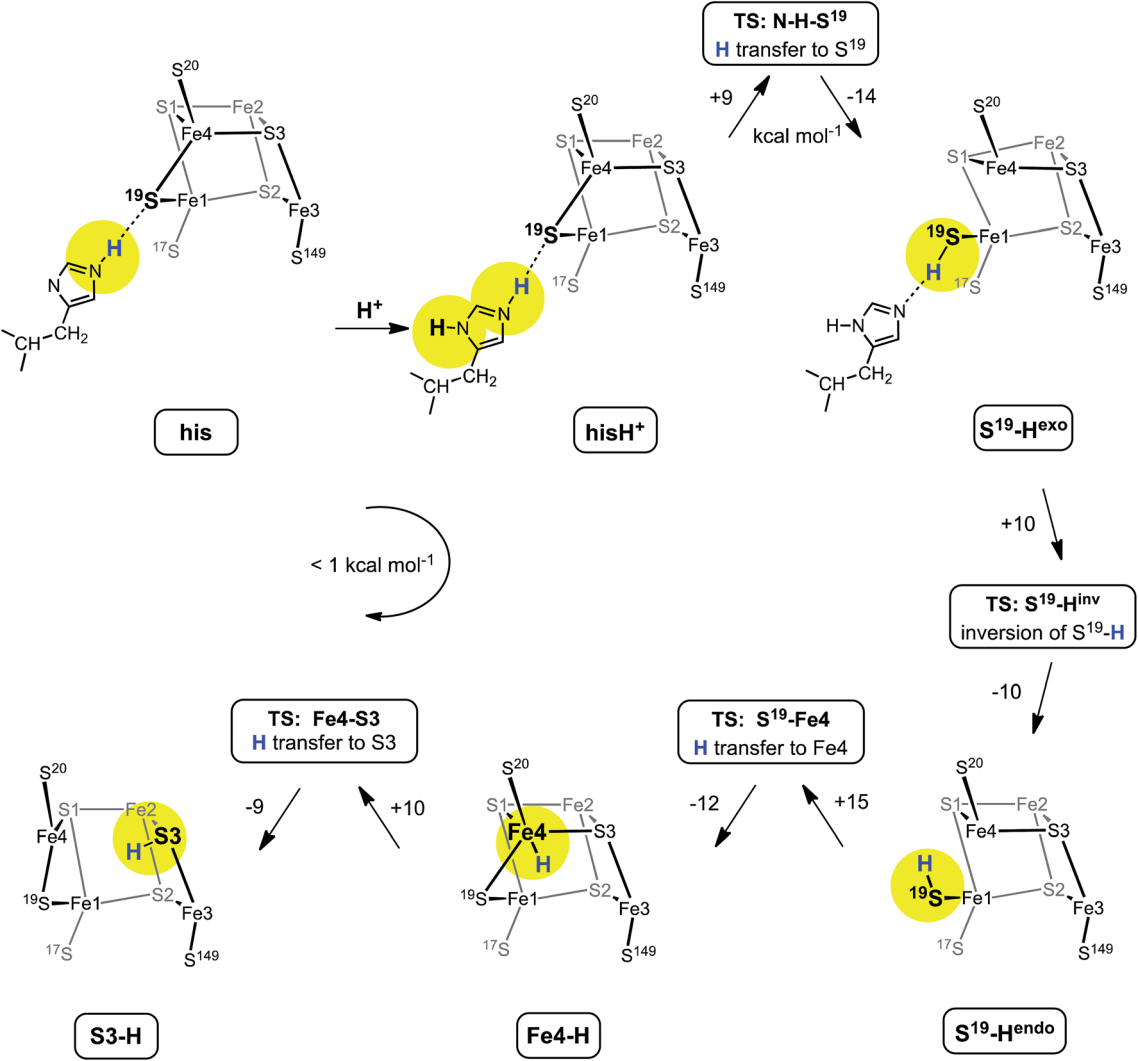
The mechanism proposed for migration of a proton from His229 to S3 in the RED state of the proximal cluster, with core [4Fe3S]^3+^. Numbers on the arrows are potential energy changes (kcal mol^–1^). Some atoms of the proximal cluster, and His229 in the last three intermediates, are not drawn.

Fig. 6 shows the mechanism calculated for the transfer of a proton from His229 to S3 of the proximal cluster in its RED state. Starting with His229 hydrogen bonded to S^19^, the Nδ atom is protonated from the proton reservoir (intermediate **hisH^+^**). The Nε proton of His229 then transfers to S^19^ across a short hydrogen bond. When S^19^ is protonated, Cys19 changes from doubly-bridging to non-bridging coordination by breaking the S^19^–Fe4 bond (**S^19^-H^exo^**). The H atom on S^19^ can then invert its configuration through a planar transition state (**S^19^-H^inv^**) to **S^19^-H^endo^**. This H atom then transfers to Fe4, and the S^19^–Fe4 bond reforms (**Fe4–H**). The final step is transfer of H to S3 (**S3–H**), with concomitant breaking of the S3–Fe4 bond. The overall H migration process is effectively energy neutral, each of the steps has an approximately symmetrical energy profile, and the intermediates are approximately equi-energetic, consistent with the requirement that this S3 protonation cycle be readily reversible. The calculated potential barriers for the steps in this mechanism, operating in both directions, range 9 to 15 kcal mol^–1^. Relatively small movements of the H atom are involved in some of the H transfer steps (*e.g.* **hisH^+^** ↔ **S^19^-H^exo^** 0.8 Å and **Fe4–H** ↔ **S3–H** 1.1 Å) and the last three steps are intramolecular, so some H atom tunneling could lower the classical barriers in this mechanism.^46^

**Scheme 2:**
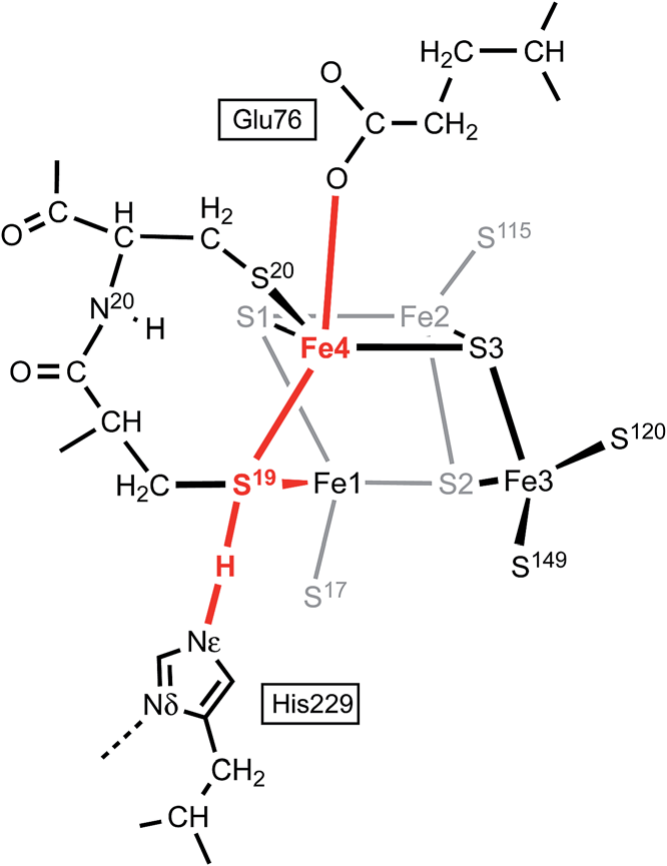
The sequence of atoms and bonds (coloured red) that is fluxional in the closed form of the proximal cluster.

In this calculated mechanistic sequence the Cys20 amide is protonated normally throughout. The three coordination of Fe4 in the calculated structure **S3–H** is completed by the N^20^ coordination that is part of the NH deprotonation cycle. The intermediates **S^19^-H^exo^** and **S^19^-H^endo^** have a long S^19^–Fe4 distance such that Fe4 is effectively three-coordinate. This would appear to be unfavourable, but the side-chain carboxylate of Glu76^S^ is adjacent to Fe4 (opposite S^19^) and readily able to complete four-coordination of Fe4 when the Fe4–S^19^ bond is extended. In the as-isolated and chemically oxidised crystals of *Ec*Hyd-1 (PDB ; 3USE, ; 3USC) the electron density was modeled with two conformations of the Glu76^S^ side-chain, one of which contains an Oε–Fe4 bond.^29^ My calculations support the feasibility of comparable movement of the Glu76^S^ side-chain in order to ligate under-coordinated Fe4, and reveal considerable fluxionality in the positions and bonding of the sequence of atoms Oε of Glu76, Fe4, S^19^, and H-Nε of His229. The entities coloured red in Scheme 2 are variable, without large barriers: thus S^19^ can lengthen one or the other of its bonds to Fe1 and Fe4; Fe4 can invert through its coordination plane (S1, S^20^, S3) towards tetrahedral stereochemistry completed by Oε of Glu76 or S^19^; and H of His229 moves to and from S^19^ as described above (see ESI Fig. S5†).

Further investigation and understanding of the mechanism will require more realistic simulations using larger computational models that combine the S3 protonation cycle and the NH deprotonation cycle, testing the nature of their synchronicity. The fluxionality shown in Scheme 2 will be part of the model. The Glu76^S^ side-chain carboxylate has dual roles in transferring the N^20^–H proton and temporarily providing additional coordination of Fe4 when it is separated from S^19^. Three events are proposed to involve Fe4: (1) the breaking and making of bonds with S^19^ and S3 during the proton migration (Fig. 6); (2) final formation of the N^20^–Fe4 bond; (3) intermediate ligation of Fe4 by Oε of Glu76^S^. Volbeda *et al.* ^29^ constructed a QM/MM model of the proximal cluster in its open SOX form, with incorporation of a considerable number of surrounding residues in a model for putative proton transfer involving Glu76. Similar hybrid QM/MM calculations are needed to evaluate the mechanistic models I propose.

### Experimental support for the proposed mechanism

The mechanism proposed relies on His229^L^ as the provider (*via* Nε) of the proton that eventually reaches S3, and also on a mechanism for compensating protonation of Nδ of His229^L^. A proton reservoir for this purpose has been identified, and the side-chain of Glu72^L^ is the mediator between the proton reservoir and Nδ of His229^L^. The recent report of the crystal structure (PDB ; 4C3O) and properties of a genetically engineered O_2_-tolerant [NiFe] hydrogenase from *Salmonella enterica* (*Se*Hyd-5) by Bowman *et al.* ^31^ provides very strong support for these ideas. These authors identified His229 as a close neighbour of the proximal cluster, conserved in all [NiFe] hydrogenases, and therefore investigated the alanine variant, finding it to be active for oxidation of H_2_, but with strongly diminished O_2_ tolerance. Bowman *et al.* also noted that Glu72^L^ (in the numbering used here: Glu73 in *Se*Hyd-5) is conserved in most O_2_-tolerant hydrogenases, but is glutamine in O_2_-sensitive hydrogenases. They tested the alanine mutant, and found it also to be compromised with respect to O_2_ tolerance. In both mutants the side-chain has no acid-base capability, and their inability to provide O_2_ tolerance is consistent with the proposed mechanism in which both are required to have proton transfer abilities. Shomura *et al.* noted that this Glu72 residue is not strictly conserved in membrane bound hydrogenases, but that the contiguous Arg (see Fig. 5) is conserved.^27^

**Fig. 7:**
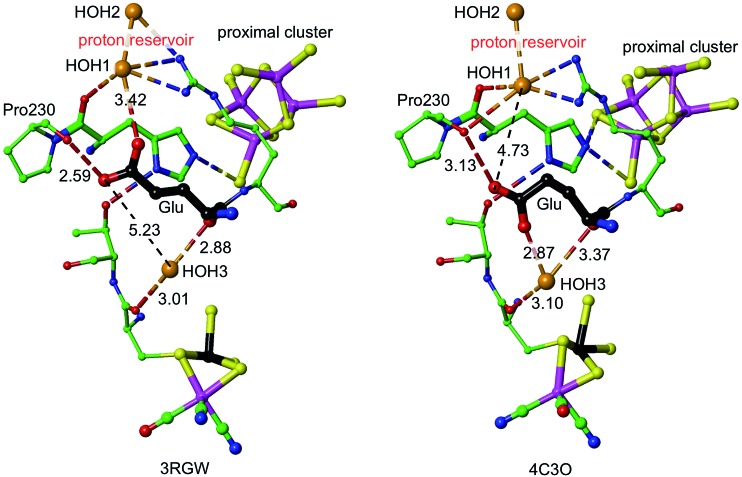
Comparative representations of the crystal structures of 3RGW (*Re*) and ; 4C3O (*Se*Hyd-5), focusing on the key His229 and Glu residues (72 in ; 3RGW, 73 in ; 4C3O, carbon atoms black) in relation to the proximal and Ni–Fe clusters (Ni black, Fe magenta), the putative proton reservoir including two water molecules, and a water molecule (HOH3) that is hydrogen bonded to O of a cysteine bridge in the NiFe site and to mainchain O of Glu. In ; 3RGW the Glu side-chain is folded up with hydrogen bonds to O of Pro230 and HOH1, while in ; 4C3O it is folded down with different hydrogen bonds to O of Pro230 and HOH3.

It is proposed that the Glu72^L^ carboxylate side-chain is mobile in transferring a proton to Nδ of His229^L^. This is supported by the observation of different conformations of this side-chain in *Re* and *Se*Hyd-5 enzymes, illustrated in Fig. 7. There is a substantial (*ca.* 2 Å) relocation of the Glu72 carboxylate O atoms, hydrogen bonding with the proton reservoir (; 3RGW), or folding down away from it (; 4C3O). The distances between the side-chain O atoms and Nδ of His229^L^ are essentially the same in these two conformations, as are the abilities of the side-chain to rotate around the Cγ–Cδ bond and transfer a proton to Nδ. The ; 4C3O crystal diffraction data yielded only 3.2 Å resolution, with only 114 water molecules located, and therefore it is significant that the water molecules shown in Fig. 7 (and in the other independent molecule) were evident and refined with below-average temperature factors. The connections shown in Fig. 7 suggest also that Glu72 and HOH3 could be part of the link that communicates O_2_-induced changes at the Ni–Fe site to the machinery for initiation of protonation of the proximal cluster.

The model suggests further experimental tests. The hydrogen bonding protonic side chain of Arg73^L^ supports the proton reservoir, and so variants without this ability are predicted to interfere with the proposed mechanism. The side-chain of Thr79^L^ hydrogen bonds to His229^L^ as part of the purported link between the Ni–Fe site and the protonation machinery at the proximal cluster: replacement with non hydrogen bonding residues could compromise the mechanism. Additional experimental tests using model systems are suggested below.

## Discussion and summary

While the relationship between the major structural changes occurring at the proximal cluster and its gating of the direction and number of electrons relayed to and from the Ni–Fe active site is understood, it is the structural change at the proximal cluster that causes the appropriate electron transfers. The structural change at the proximal cluster is not simply a consequence of its change in redox state, but is the instigator of redox change. Change in geometrical structure from RED to SOX modifies also the electronic structure and the redox potentials, such that the proximal cluster can readily release two electrons to the active site where they are required to rescue the Ni–Fe cluster from its O_2_-induced forms that are inactive in the normal H_2_ oxidation cycle.

Therefore the chemical cause of the structural change RED → SOX at the proximal cluster must be questioned, and this report provides a response. It is proposed that there is a closed proton shuttle involving the μ_3_-S3 atom, which, when protonated to become μ-SH, breaks its bond with Fe4 and facilitates the alternative coordination of Fe4 by the ‘hard’ deprotonated backbone amide N^20^, concomitant with the movement of the N^20^ proton to the side-chain of Glu76, as described by Pelmenschikov and Kaupp.^44^ The idea that protonation of cluster sulfide causes disruption of cluster structure evolved from investigations of the effects of protonation of [Fe_4_S_4_X_4_] clusters^42,43^ and of simulations of similar steps at the FeMo-co cluster of nitrogenase.^41^ An H atom on S3 would not have been detected in the diffraction analyses of the SOX form of the proximal cluster.

It is proposed that the proton reaching S3 is from His229-Nε (or possibly from a water molecule in the vicinity of His229), being transferred first to the S of Cys19, then to Fe4, and then to S3. The potential energy profile for this trajectory of H over the RED form of the proximal cluster is accessible. Other trajectories are conceivable, but, at this stage, appear to be less favourable energetically. Both proton movements at the proximal cluster, the NH deprotonation cycle and the S3 protonation cycle, are reversible and involved in the RED ↔ SOX interconversion. They are expected to be synchronous, but details of the sequence of events in this double proton shuttle are still to be explored. Pandelia *et al.* ^32^ reported pH dependence of the RED/OX potential, with a coupled p*K*_a_ of 6.9–7.1, but the identity of the species involved is unknown.

The initial proton transfer from Nε-H of His229 to S^19^ requires assistance through protonation of the Nδ atom of His229. A mechanism for this has been identified, involving a nearby ‘proton reservoir’ involving residues Glu72^L^, Arg73^L^, and associated water molecules. Reconformation of the acidic side-chain of Glu72^L^ (already evident in some crystal structures) is able to deliver the proton required at His229-Nδ. This aspect of the overall mechanism is supported by some experimental observations. His229^L^ is a fully conserved residue, and Glu72^L^ is highly conserved. Mutation of either residue to alanine with a proton-inert side-chain compromises the O_2_-tolerance of the enzyme.^31^

One more element is required for this model, namely communication from the Ni–Fe site when it is subject to O_2_. There is a conserved link from one of the cysteines bridging Ni and Fe, Cys78^L^, through Thr79^L^ which hydrogen bonds to His229-Nδ. Further, Glu72^L^ is hydrogen bonded through a water molecule to CO of Cys78^L^. Thus, geometrical changes in the bridging region of the Ni–Fe catalytic site, caused by O_2_ which is believed to bind there, can be directly communicated to the His229^L^ + Glu72^L^ molecular machinery that initiates protonation of the proximal cluster.

In summary, the complete sequence of events involved in the triggering of O_2_ tolerance is envisioned as: (1) binding of O_2_ at the Ni–Fe bridge; (2) conformation changes transmitted through Cys78^L^ and Thr79^L^ to the Glu72^L^–Agr73^L^–water proton reservoir, which then (3) protonates His229-Nδ; (4) being thereby acidified, His229-Nε-H protonates S^19^ of the unique bridging cysteine of the proximal cluster in its RED state; (5) trigonal pyramidal S^19^–H inverts, to point towards Fe4, and transfers H to Fe4; (6) Fe4 (which is five-coordinate at this point), transfers H to S3 along the Fe-4–S3 bond, resulting in doubly-bridging S3–H which moves well away from Fe4; (7) at approximately the same time the N^20^–H proton is abstracted by the carboxylate side-chain of Glu76^L^, which is in proximity to Fe4 and could ligate Fe4 at earlier stages; (8) these changes in the geometric and electronic structure of the proximal cluster to that of the SOX state change its redox potential such that it can release two electrons to the Ni–Fe site and initiate the reduction of the O_2_-inactivated forms of the catalytic site; (9) on release of the two electrons from the proximal cluster in the SOX geometry and bonding configuration, the proton flow is fully reversed as the proximal cluster recovers its RED geometry and bonding configuration to match its RED electron count. Protons involved at the Ni–Fe catalytic site, in either the reduction of O_2_ (to OH_2_) or oxidation of H_2_, are postulated to be independent of the proton cycles that involve the proximal cluster. In the absence of O_2_ none of this molecular machinery and major geometrical change is needed, because the proximal cluster functions simply as a one-electron transfer agent involving the RED and OX states with minor geometrical adjustment.

This theory could be tested with model systems. When a synthetic cluster [Fe_4_S_3_(SR)_6_]^*z*^ becomes available, cyclic voltammetric measurements of the *z* = –3/–2 and –2/–1 steps are expected to be informative. With aprotic conditions, it is predicted that standard well-separated potentials will be observed, without coupled chemical steps. Then, with stoichiometrically controlled addition of a non-coordinating Bronsted acid, it is predicted that the potentials and their separation will change, and that there will be coupled chemical events indicative of geometrical change. The expected slower rates of coupled geometrical changes, and possibly their restricted reversibility, are measureable in principle. Further, with controlled addition of a suitable ligand (non Bronsted basic), an intermediate analogous to the SOX form of the proximal cluster could be generated, to some extent replicating the chemistry (ie combination of proton transfer plus electron transfer plus ligation) proposed for the proximal cluster of O_2_-tolerant [NiFe] hydrogenases. However, there are complications arising from the alternative μ_3_-S protonation sites available in [Fe_4_S_3_(SR)_6_] and alternative Fe–S(H) extensions.^47^ In the absence of a [Fe_4_S_3_(SR)_6_]^*z*^ model, similar experiments with readily available [Fe_4_S_4_(SR)_4_]^*z*^ systems could be informative, see Fig. 3, remembering that the O_2_ sensitive enzymes possess a [Fe_4_S_4_(SR)_4_] cluster at the proximal position. There is a caveat here, because these model systems in solution are subject to acid-catalysed ligand substitution reactions^42,43^ that are unlikely to occur in the protein.

### Methods

All calculations reported in this paper use Delley's DMol3 package,^48–50^ with numerical basis sets (dnp). The calculations are all-electron and spin unrestricted. The geometric models and the electronic states, together with method validation results and functional choice, are fully described in the ESI.†
