# Supplementary material for: What is the trigger mechanism for the reversal of electron flow in oxygen-tolerant [NiFe] hydrogenases?
Source: Chem Sci. 2014 Dec 8;6(2):1433–43. doi: 10.1039/c4sc03223c (PMC5811149; doi:10.1039/c4sc03223c)
Supplement: Supplementary file 1 [file SC-006-C4SC03223C-s001.pdf]

Electronic Supplementary Material (ESI) for Chemical Science.
This journal is © The Royal Society of Chemistry 2014

What is the trigger mechanism for the reversal of electron flow in oxygen-tolerant hydrogenases?

Ian Dance

School of Chemistry, University of New South Wales, Sydney 2052, Australia. e-mail: i.dance@unsw.edu.au

Supplementary information

Computational methods

All calculations reported in this paper use Delley's DMol3 package,[1-3] with numerical basis sets (dnp). The calculations are all-electron, spin unrestricted, with a fine integration mesh. Validation results have been reported[4-7] (and see below). Potential energy surfaces around the intermediates and reaction trajectories were explored. Transition states were located and checked using the method previously developed for the complex trajectories of reactions of the FeMo-co cluster in nitrogenase.[8,9]

The basic models used are shown in Fig S1. Model (a) includes residues 17, 18, 19, 20, 113, 114, 115, with cysteines 120 and 149 truncated as $SCH_2C^{\alpha}H_3$ with the C^{α} atom fixed, and with the C^{α} atom of His229 capped as CH_3. The location of His229 C^{α} was monitored and sometimes fixed, allowing conformational variability of the His229 sidechain, without unreasonable positional excursions. Once it was demonstrated that the hydrogen bonds from N^{114}-H and N^{115}-H to S3 did not control the position of S3 (although they prevent *exo* protonation of S3), this section was not included and cysteine 115 was treated as $SCH_2C^{\alpha}H_3$ with the C^{α} atom fixed (Fig S1 (b)). Models (a) and (b) represent the RED state, with core charge $[Fe_4S_3]^{3+}$. Model (c) and variants was used in explorations of water coordinated to Fe1.

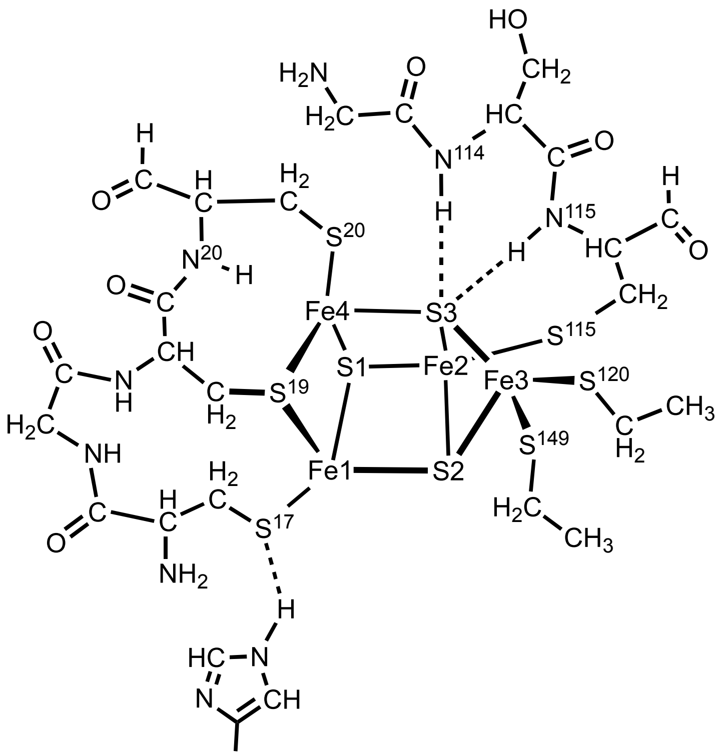

(a)

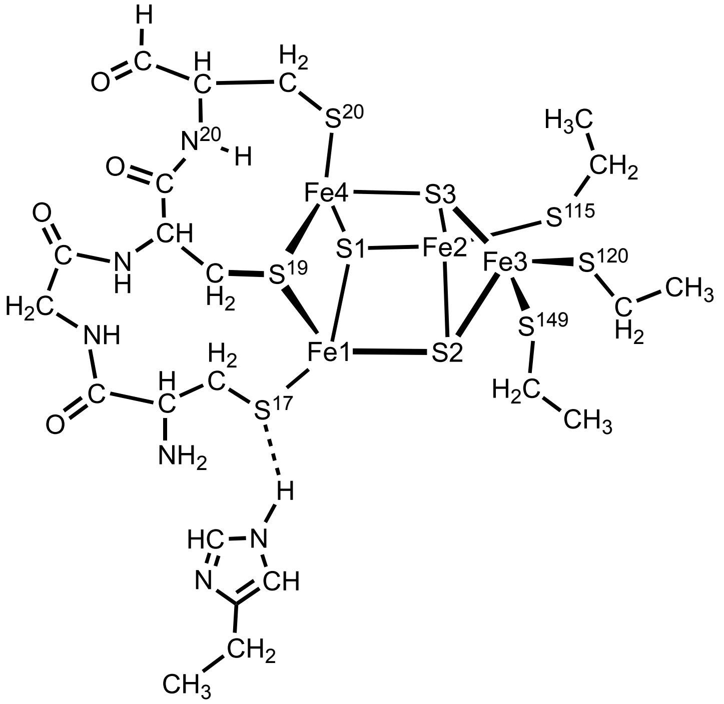

(b)

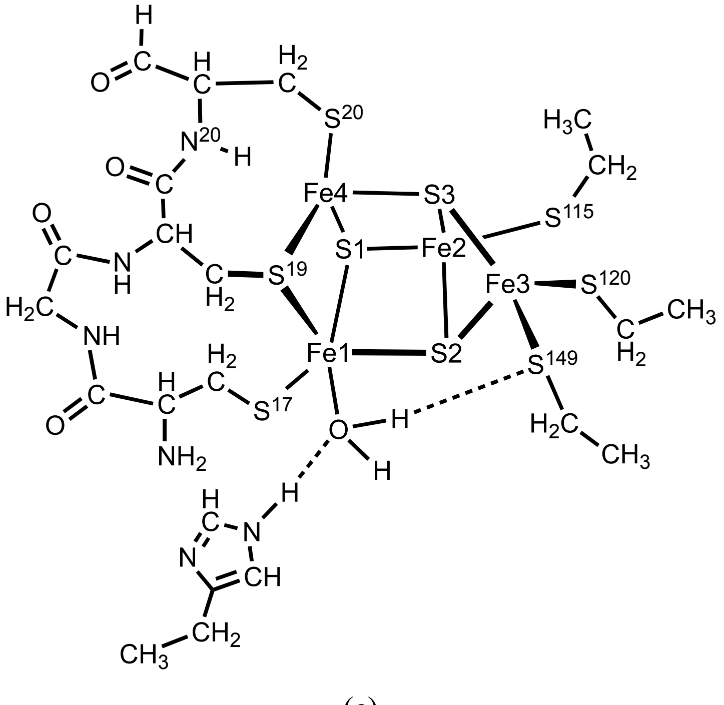

(c)

Fig. S1.

Electronic state. As in previous calculations, the electronic states of models were specified and controlled by fixing the signs of the spin densities on the four Fe atoms, and the total spin state S. Some exploration of the relative energies of electronic states was undertaken, mainly with $[Fe_4S_2(SH)(SMe)_6]^{2-}$, that is $[Fe_4S_3(SMe)_6]^{3-}$ protonated in the *endo* configuration with the same geometry as the protonated form of S3 in the proximal cluster. The energy variations were minor, with the Fe1 +, Fe2 -, Fe3 +, Fe4 -, S = -1/2 state being ca 1 kcal mol^{-1} more stable than Fe1 +, Fe2 +, Fe3 -, Fe4 -, S = 1/2 state (Fe atom numbering as in Fig. S1). These are the BS13, BS34 states in the nomenclature of previous authors.[10,11] The structures calculated for the protonation mechanism (Fig. 6, and presented below) were all on the Fe1 +, Fe2 +, Fe3 -, Fe4 -, S = -1/2 electronic surface (BS12). Magnitudes of the Fe spin densities are generally ca 3.1. According to Pandelia *et al* the RED state has S = 1/2.[12] Previous authors have discussed the electronic states of the proximal cluster

in some detail, in relation to spectroscopic data.[10,13,12,14,11] In my experience with DFT calculations on FeS clusters, the shapes of the potential energy surfaces, and thus the calculated barriers for reaction steps, are only slightly dependent on the electronic state followed.

Two density functionals were tested, through calculations of the RED structure, PDB 3RGW, using a model that contained the six cysteine residues in full. Comparison of calculated bond distances are presented in Table S1. The pbe distances are shorter than those of blyp and are closer to the experimental distances. Pelmenschikov [15] also found pbe to give tighter binding than b3lyp. In the models presented here Cys19 is sometimes protonated, which is expected to weaken its coordination, and it also unbridges. The pbe functional was found to give more realistic distances for such Fe-S(H)-cys coordination.

Table S1. Comparison of calculated and experimental bond distances in the RED form of the proximal cluster.

bond	experimental (PDB 3RGW)	calculated blyp (BS24)	calculated pbe (BS24)
Fe1-S19	2.31	2.40	2.33
Fe4-S19	2.31	2.42	2.35
Fe1-S1	2.29	2.32	2.29
Fe1-S2	2.27	2.40	2.35
Fe2-S1	2.28	2.33	2.29
Fe2-S2	2.28	2.28	2.25
Fe2-S3	2.27	2.33	2.29
Fe2-S15	2.27	2.35	2.31
Fe3-S2	2.29	2.34	2.30
Fe3-S3	2.29	2.26	2.22
Fe3-S120	2.34	2.40	2.36
Fe3-S149	2.30	2.34	2.30
Fe4-S1	2.28	2.32	2.27
Fe4-S3	2.31	2.40	2.35
Fe4-S20	2.30	2.36	2.31
	jmol_3RGW_fe4s3_mdl _140801	3grwh.car_11	3grwhpbe.car_3

Protonation of the $[Fe_4S_3(SR)_6]$ structure type.

Protonation of an $[Fe_4S_3(SR)_6]$ cluster could occur at the thiolate ligand and/or μ_3-S, and I have investigated aspects of both. As expected, protonation of SMe elongates its bond(s) to Fe. Assuming C_s symmetry for the core of $[Fe_4S_3(SMe)_6]^{3-}$ there are ten ways to protonate μ_3-S and elongate a bond of the resulting μ-SH to Fe: the μ-SH function bridging two Fe atoms has pyramidal stereochemistry, and there are two configurations for the S-H bond, either *endo* or *exo* relative to the elongated S-Fe bond. Detailed results from these calculations, including the effects of electronic structure, will be described separately, but the outcome relevant to the present context is shown in Fig S2: protonation of the S atom homologous to S3 of the proximal cluster causes the S3-Fe4 bond to break (to 3.28Å), and the structural change is reversed on deprotonation.

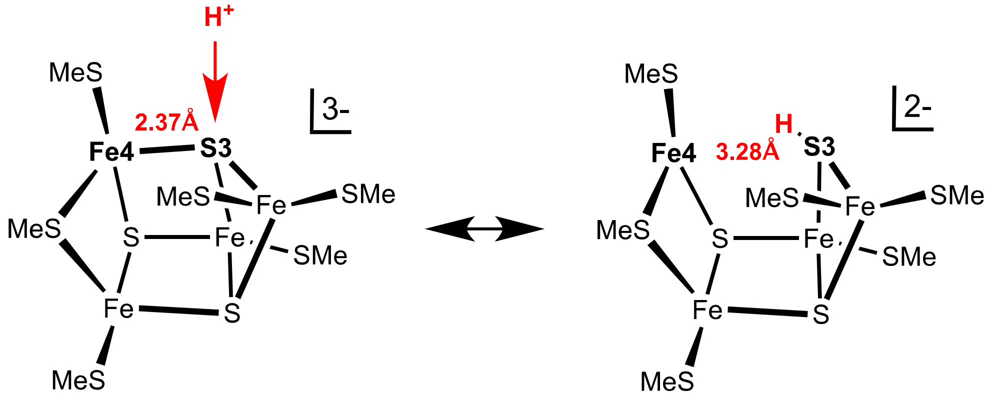

Fig S2. The consequences of protonation of S3 in $[Fe_4S_3(SMe)_6]^{3-}$. The S3-H conformation is *endo*, towards Fe4.

The NH deprotonation cycle

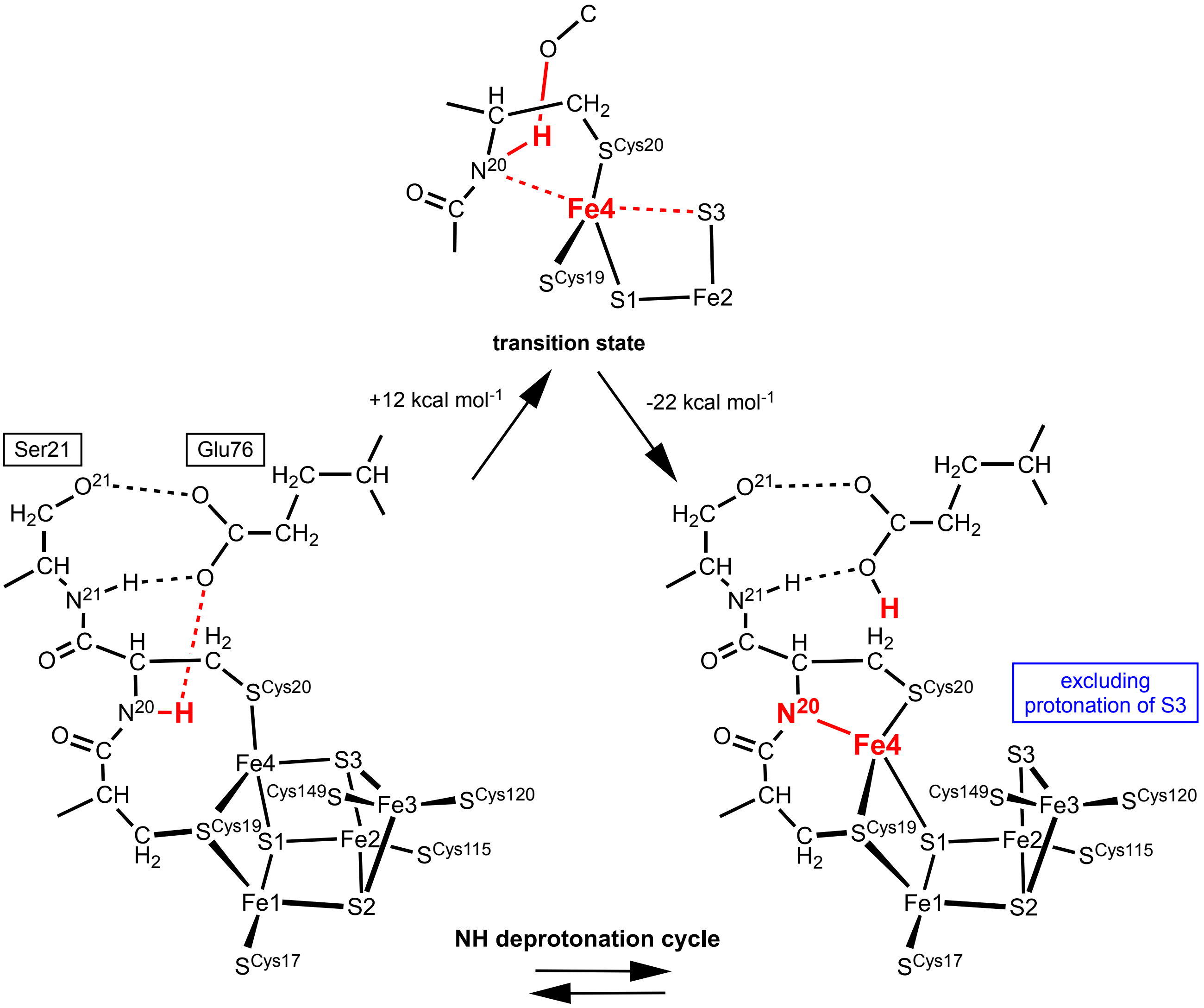

Fig. S3. The essence of the mechanism calculated by Pelmenschikov and Kaupp[15] for the movement of the proton on N^{20} to the side-chain of Glu76 of the super-oxidised form of the proximal cluster $[4Fe\text{-}3S]^{5+}$, and formation of the N^{20}-Fe4 bond (adapted from reference [15]). This is the NH deprotonation cycle.

Oxidation of the closed form of the proximal cluster.

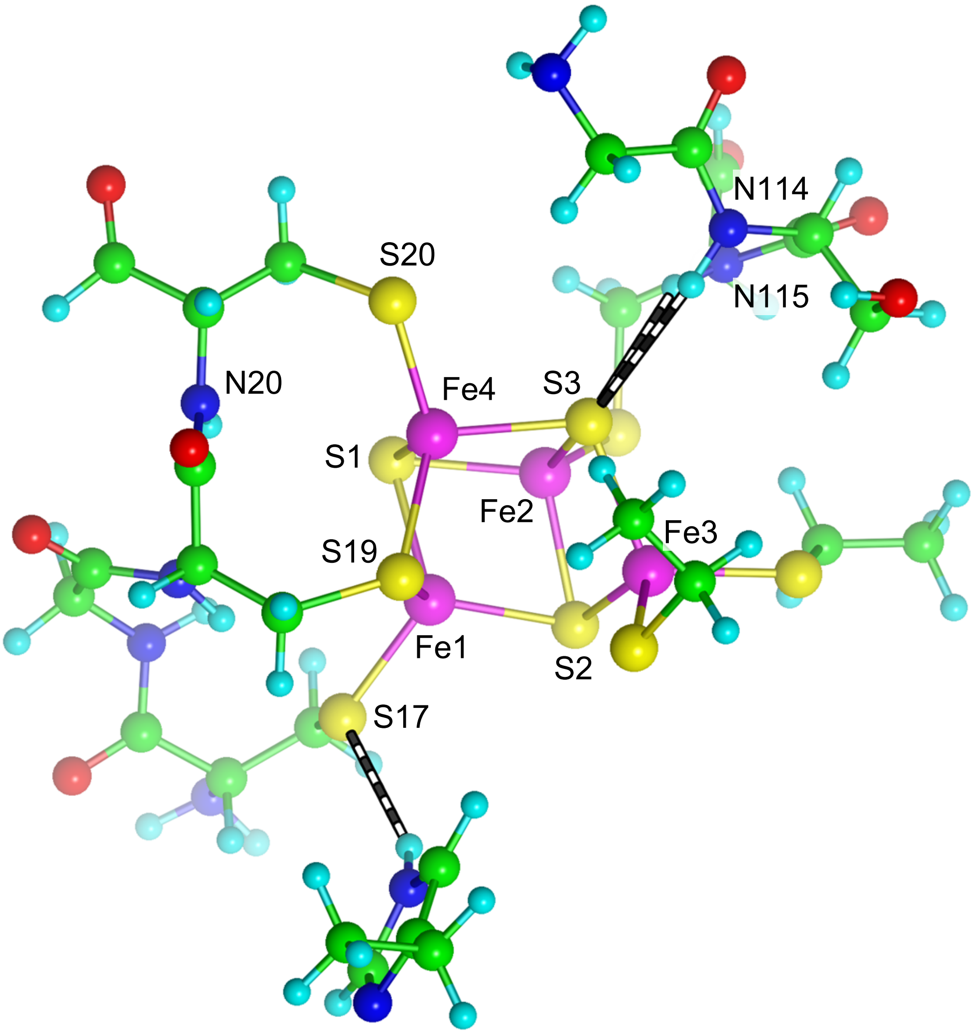

Fig S4. Model used for calculation of the redox states of the closed cluster. Residues 17, 18, 19, 20, 115, 114 are included in full: residues Cys120, Cys149 and His229 are capped as CH_3 at the Cα atom. Broken bonds are N-H--S hydrogen bonds.

Table S2. Atom properties for three oxidation states of the model in Fig S3.

	RED core $[Fe_4S_3]^{3+}$		OX core $[Fe_4S_3]^{4+}$		SOX core $[Fe_4S_3]^{5+}$	
atom	charge	spin density	charge	spin density	charge	spin density
Fe1	0.57	2.87	0.61	3.19	0.59	3.06
Fe2	0.60	3.02	0.60	3.09	0.56	2.79
Fe3	0.71	-3.40	0.70	-3.36	0.65	-3.10
Fe4	0.57	-2.97	0.54	-2.70	0.61	-3.16
S1	-0.70		-0.62		-0.54	
S2	-0.60		-0.55		-0.48	
S3	-0.74		-0.68		-0.62	
S17	-0.66		-0.61		-0.53	
S19	-0.45		-0.40		-0.38	
S20	-0.53		-0.46		-0.40	

Table S3. Selected distances (Å) for three oxidation states of the model in Fig S3.

	RED<br>core $[Fe_4S_3]^{3+}$	OX<br>core $[Fe_4S_3]^{4+}$	SOX<br>core $[Fe_4S_3]^{5+}$
S3-Fe2	2.28	2.27	2.25
S3-Fe3	2.4386	2.42	2.42
S3-Fe4	2.42	2.40	2.36
S1-Fe1	2.36	2.36	2.34
S1-Fe2	2.34	2.34	2.30
S1-Fe4	2.28	2.25	2.25
S2-Fe1	2.35	2.34	2.32
S2-Fe2	2.35	2.34	2.32
S2-Fe3	2.25	2.24	2.21
S19-Fe1	2.38	2.38	2.35
S19-Fe4	2.41	2.36	2.43

Coordination of Glu76 to Fe4

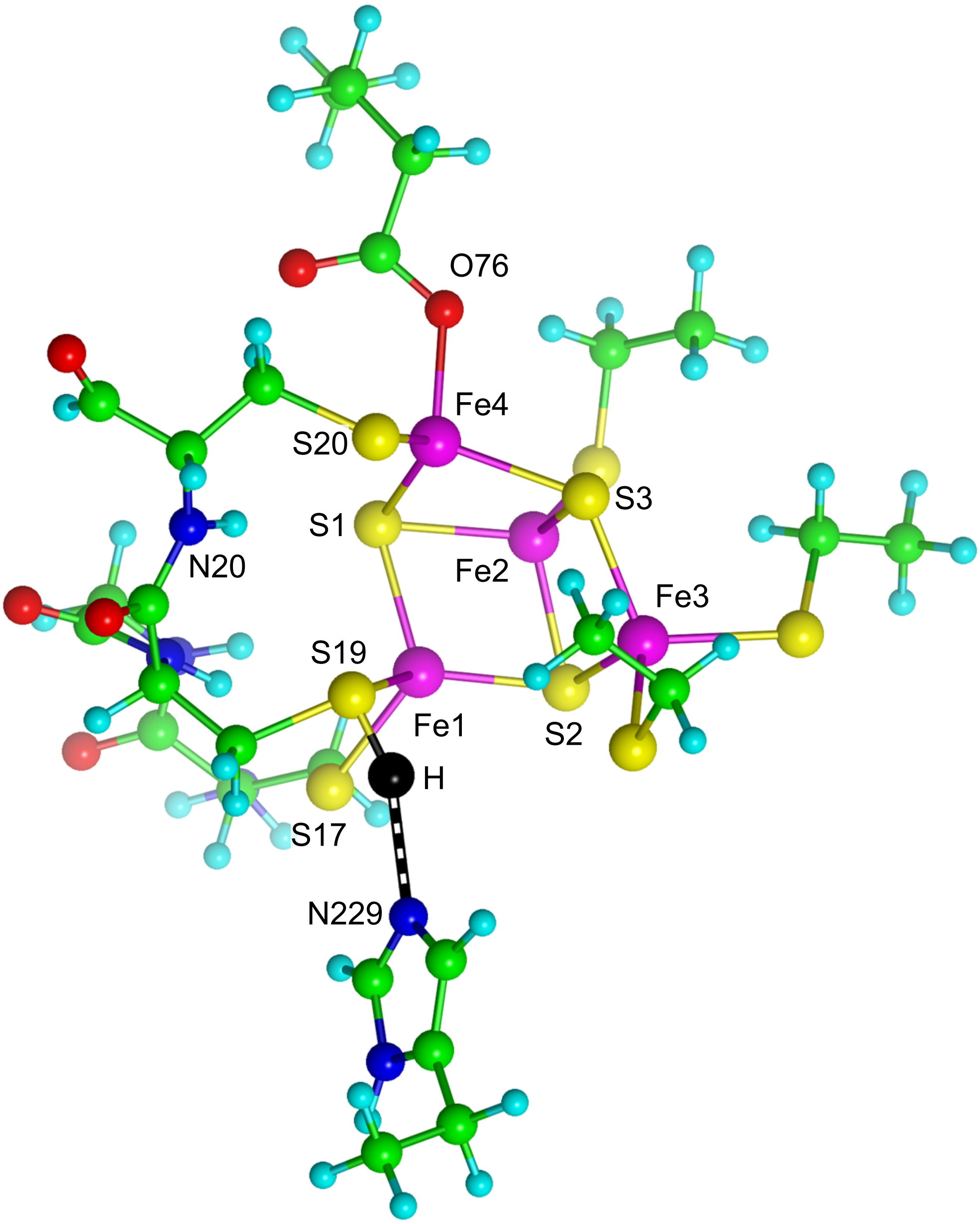

Fig S5. Model of **S19-H^{exo}** with coordination of Glu76 side chain to Fe4.

Water coordination of Fe1

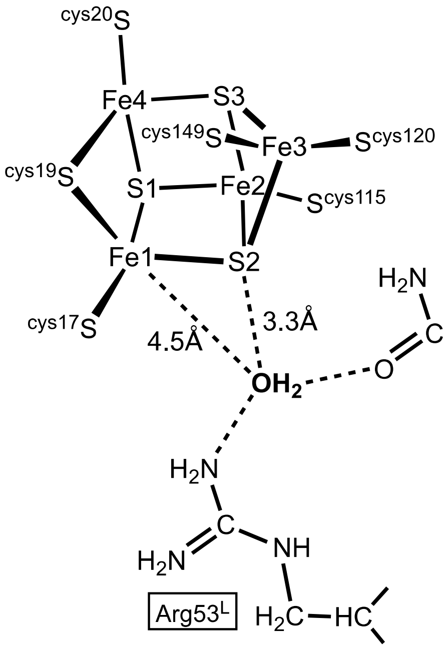

Fig. S6. A conserved water molecule hydrogen bonded to Gln122^{S} and Arg53^{L} could move to Fe1.

Table S4. Pictures and cartesian coordinates (Å) for the calculated structures in Fig. 6.

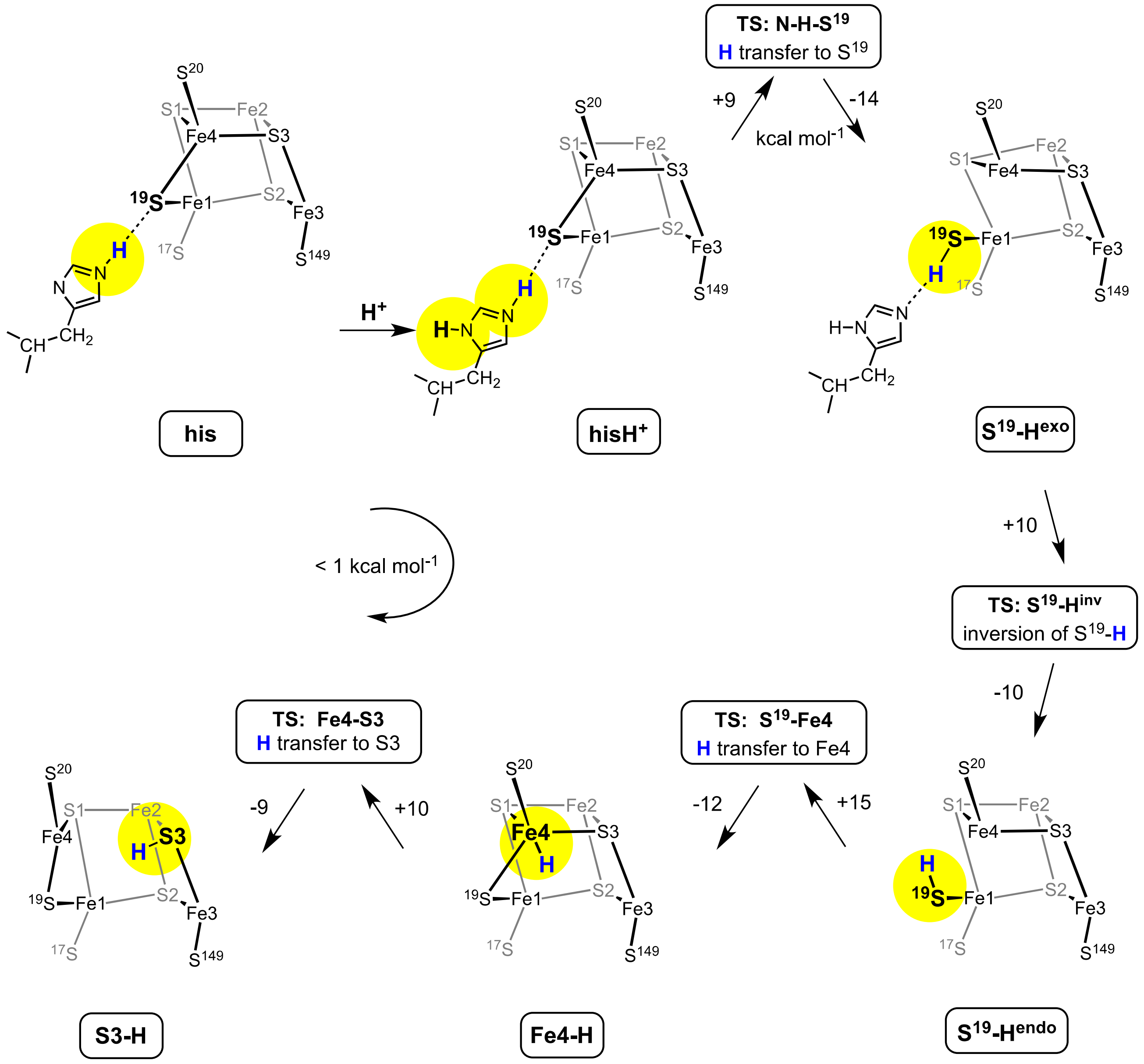

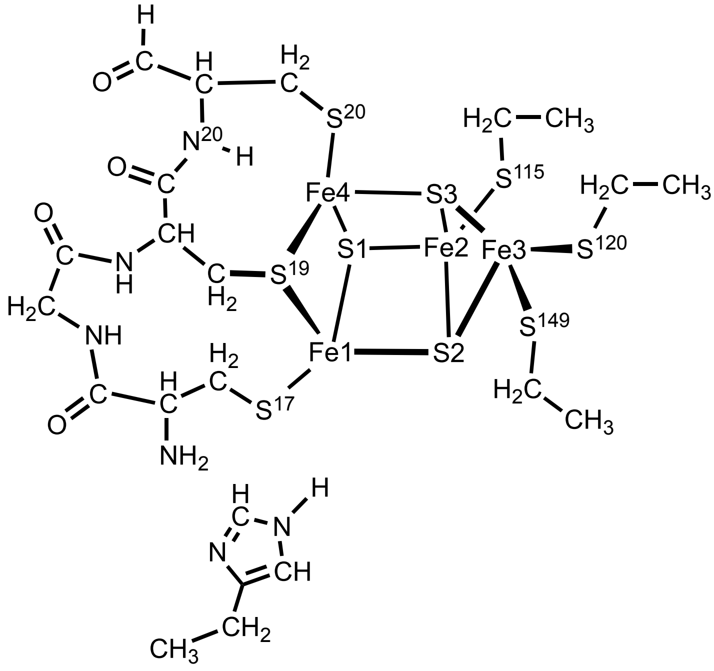

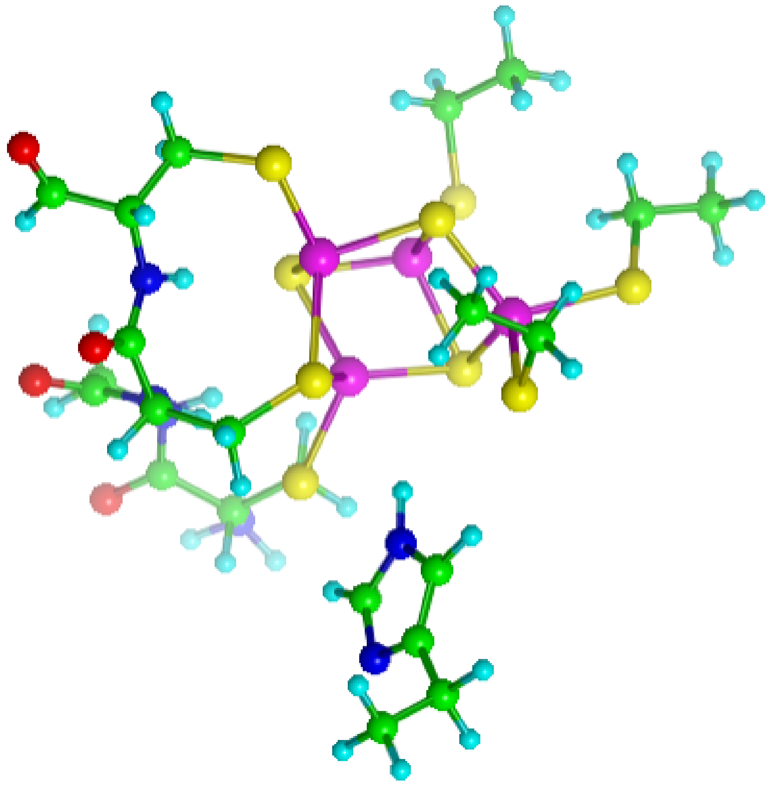

redv318n19pb_4a.cmmf

his

85				
C	1	7.503	12.581	9.891
C	2	8.650	13.178	9.062
C	3	8.196	14.199	8.062
C	4	8.531	15.538	7.986
C	5	7.167	14.995	6.341
C	6	5.947	17.270	1.271
C	7	3.241	18.568	5.971
C	8	2.737	19.819	6.711
C	9	4.627	18.123	6.478
C	10	2.784	22.262	6.815
C	11	1.318	22.627	6.688
C	12	3.647	23.379	6.186
C	13	9.967	24.868	2.539
C	14	8.742	24.250	1.867
C	15	13.683	22.348	2.872
C	16	12.315	22.248	3.551
C	17	9.201	20.422	8.998
C	18	10.452	19.925	8.280
C	19	4.685	16.631	0.694
C	20	3.349	17.349	0.948
C	21	2.206	19.058	2.301
C	22	2.080	18.916	3.829
Fe	23	8.089	21.390	3.293
Fe	24	9.959	20.286	5.030
Fe	25	6.866	19.155	3.908
Fe	26	6.326	21.400	5.327
H	27	10.868	24.737	1.918
H	28	9.814	25.950	2.721
H	29	9.396	13.642	9.730
H	30	1.329	18.581	1.848
H	31	9.190	16.180	8.563
H	32	7.861	11.785	10.567
H	33	7.015	13.360	10.497
H	34	6.742	12.158	9.219
H	35	7.892	17.006	6.572
H	36	6.574	15.119	5.438
H	37	9.170	12.358	8.533
H	38	6.823	16.709	0.909
H	39	4.086	18.384	4.024
H	40	6.071	18.305	0.924
H	41	3.343	24.329	6.648
H	42	3.437	23.439	5.105
H	43	3.028	22.183	7.885
H	44	0.787	22.104	5.853
H	45	2.524	17.785	6.278
H	46	4.559	18.043	7.571
H	47	4.830	17.121	6.075
H	48	13.902	21.430	2.304
H	49	4.548	15.665	1.228
H	50	2.162	20.139	2.089
H	51	13.710	23.206	2.175
H	52	8.584	19.579	9.345
H	53	4.299	18.782	2.060
H	54	8.583	21.020	8.314
H	55	3.883	16.103	-1.089
H	56	9.467	21.050	9.871
H	57	5.480	15.714	-0.960
H	58	11.083	19.333	8.968
H	59	11.060	20.769	7.920
H	60	14.486	22.479	3.618
H	61	12.098	23.188	4.082
H	62	11.524	22.114	2.799
H	63	7.863	24.356	2.521
H	64	8.513	24.775	0.922
H	65	3.744	21.013	5.383

H	66	10.144	24.376	3.504
N	67	7.340	13.866	7.026
N	68	7.868	16.027	6.886
N	69	3.052	21.007	6.140
N	70	3.227	18.685	4.517
N	71	4.795	16.451	-0.774
N	72	3.389	18.478	1.699
O	73	2.112	19.715	7.777
O	74	0.746	23.458	7.387
O	75	2.300	16.869	0.474
O	76	0.971	19.091	4.359
S	77	5.964	17.204	3.133
S	78	6.190	19.111	6.131
S	79	5.463	23.167	6.463
S	80	8.961	22.457	1.448
S	81	12.223	20.860	4.773
S	82	10.091	18.832	6.827
S	83	9.059	19.311	3.249
S	84	8.554	22.121	5.352
S	85	5.804	21.103	3.157

Key H atoms are emphasised as large black spheres.

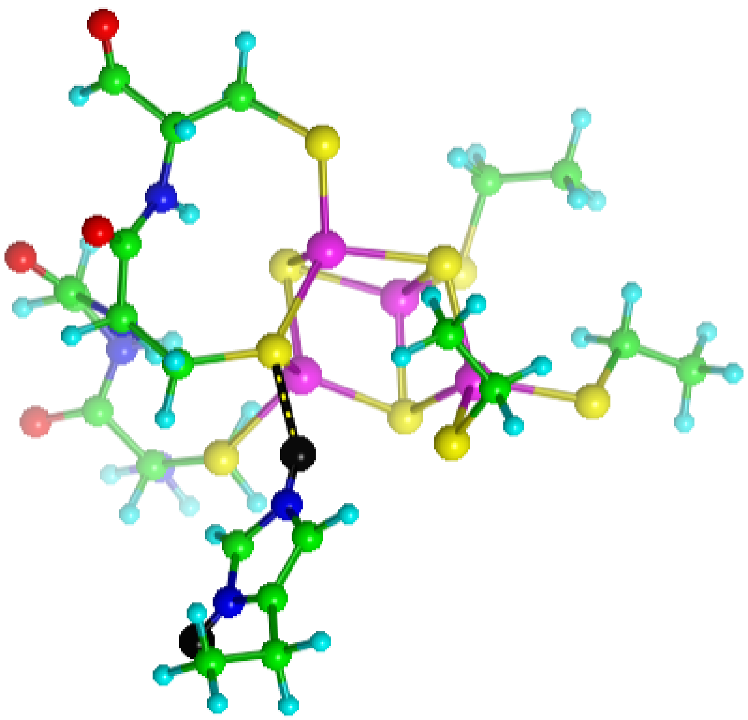

redv318nh19hpbtg_12.cmmf

hisH+

86

C	1	12.266	9.551	8.210
C	2	13.535	9.702	7.352
C	3	13.463	10.775	6.313
C	4	14.135	11.962	6.178
C	5	12.813	11.797	4.408
C	6	12.532	13.458	-0.358
C	7	10.561	16.173	4.073
C	8	10.642	17.591	4.667
C	9	11.650	15.267	4.673
C	10	11.605	19.831	4.445
C	11	10.423	20.751	4.200
C	12	12.853	20.420	3.752
C	13	19.133	18.923	-0.147
C	14	17.648	18.803	-0.481
C	15	21.549	15.177	0.504
C	16	20.249	15.664	1.161
C	17	16.886	15.872	6.759
C	18	17.789	14.799	6.160
C	19	11.156	13.316	-0.997
C	20	10.112	14.393	-0.655
C	21	9.661	16.582	0.365
C	22	9.551	16.683	1.894
H	23	12.005	9.938	4.950
H	24	13.969	13.562	4.700
Fe	25	15.943	16.550	1.148
Fe	26	17.378	14.963	2.900
Fe	27	14.032	15.054	2.045
Fe	28	14.507	17.485	3.195
H	29	19.742	18.413	-0.911
H	30	19.446	19.983	-0.100
H	31	14.390	9.935	8.004
H	32	8.658	16.372	-0.021
H	33	14.921	12.405	6.775
H	34	12.399	8.754	8.956
H	35	12.029	10.479	8.751
H	36	11.381	9.288	7.610
H	37	12.362	11.998	3.428
H	38	13.773	8.735	6.869
H	39	13.170	12.617	-0.670
H	40	11.220	15.472	2.163
H	41	13.041	14.373	-0.690
H	42	12.958	21.461	4.091
H	43	12.710	20.428	2.659
H	44	11.781	19.784	5.530

	H	45	9.746	20.423	3.371
	H	46	9.591	15.803	4.455
	H	47	11.567	15.354	5.766
	H	48	11.404	14.229	4.396
	H	49	21.411	14.176	0.064
	H	50	10.700	12.386	-0.599
	H	51	9.967	17.579	0.011
	H	52	21.853	15.874	-0.297
	H	53	15.984	15.428	7.210
	H	54	11.558	15.701	0.015
	H	55	16.554	16.577	5.984
	H	56	10.346	13.146	-2.866
	H	57	17.422	16.441	7.543
	H	58	11.868	12.529	-2.758
	H	59	18.139	14.099	6.939
	H	60	18.681	15.250	5.700
	H	61	22.367	15.112	1.240
	H	62	20.406	16.661	1.599
	H	63	19.448	15.765	0.414
	H	64	17.040	19.256	0.315
	H	65	17.414	19.330	-1.420
	H	66	11.860	18.171	3.126
	H	67	19.348	18.455	0.822
	N	68	12.641	10.695	5.184
	N	69	13.728	12.559	5.010
	N	70	11.335	18.499	3.941
	N	71	10.555	16.117	2.617
	N	72	11.276	13.313	-2.473
	N	73	10.560	15.576	-0.157
	O	74	10.119	17.836	5.762
	O	75	10.224	21.792	4.811
	O	76	8.912	14.155	-0.871
	O	77	8.623	17.330	2.399
	S	78	12.469	13.468	1.494
	S	79	13.476	15.516	4.271
	S	80	14.426	19.531	4.150
	S	81	17.107	17.045	-0.739
	S	82	19.646	14.549	2.520
	S	83	16.952	13.777	4.858
	S	84	16.040	14.281	1.257
	S	85	16.814	17.228	3.072
	S	86	13.726	17.163	1.109

	86				
	C	1	12.814	8.675	8.068
	C	2	13.903	8.992	7.022
	C	3	13.602	10.134	6.096
	C	4	14.209	11.352	5.875
	C	5	12.545	11.239	4.437
	C	6	12.519	13.529	-0.782
	C	7	10.671	16.030	3.840
	C	8	10.839	17.423	4.492
	C	9	11.618	14.973	4.438
	C	10	11.785	19.672	4.363
	C	11	10.581	20.603	4.263
	C	12	12.986	20.334	3.662
	C	13	19.145	18.891	-0.876
	C	14	17.646	18.861	-1.192
	C	15	21.587	15.122	-0.537
	C	16	20.291	15.571	0.157
	C	17	17.397	15.598	6.065
	C	18	18.222	14.524	5.364
	C	19	11.133	13.408	-1.411
	C	20	10.059	14.389	-0.908
	C	21	9.589	16.528	0.195
	C	22	9.599	16.606	1.729

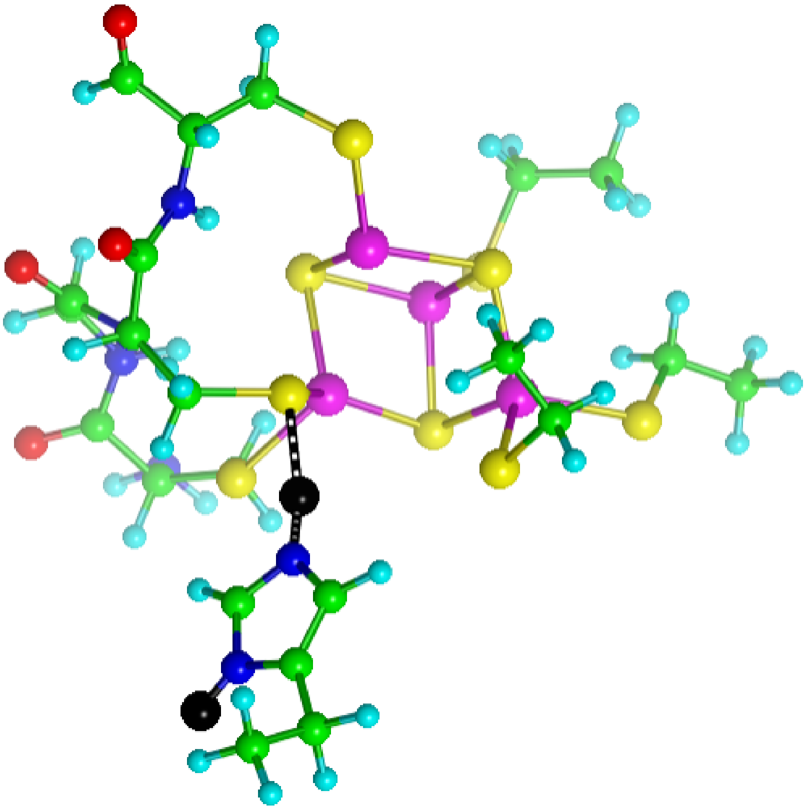

redv318nh19hpbts_1_8778.16.cmmf

TS: N-S19

H	23	11.903	9.311	5.051
H	24	13.656	13.131	4.525
Fe	25	15.996	16.574	0.619
Fe	26	17.571	14.908	2.177
Fe	27	14.135	14.946	1.711
Fe	28	14.862	17.553	2.809
H	29	19.718	18.327	-1.630
H	30	19.519	19.931	-0.851
H	31	14.848	9.221	7.536
H	32	8.570	16.252	-0.102
H	33	15.093	11.782	6.334
H	34	13.120	7.818	8.686
H	35	12.640	9.532	8.733
H	36	11.855	8.412	7.599
H	37	11.878	11.463	3.607
H	38	14.101	8.079	6.427
H	39	13.147	12.710	-1.165
H	40	11.297	15.399	1.886
H	41	13.006	14.465	-1.090
H	42	13.084	21.347	4.077
H	43	12.789	20.428	2.583
H	44	12.014	19.554	5.433
H	45	9.829	20.324	3.482
H	46	9.660	15.753	4.186
H	47	11.548	15.101	5.525
H	48	11.204	13.990	4.180
H	49	21.496	14.088	-0.904
H	50	10.720	12.414	-1.146
H	51	9.790	17.547	-0.173
H	52	21.813	15.780	-1.395
H	53	16.496	15.166	6.531
H	54	11.496	15.742	-0.323
H	55	17.059	16.354	5.342
H	56	10.357	13.325	-3.312
H	57	17.989	16.107	6.848
H	58	11.974	13.012	-3.244
H	59	18.572	13.767	6.086
H	60	19.110	14.960	4.886
H	61	22.439	15.153	0.162
H	62	20.417	16.595	0.535
H	63	19.456	15.597	-0.557
H	64	17.080	19.388	-0.411
H	65	17.449	19.377	-2.146
H	66	12.004	18.091	2.937
H	67	19.328	18.434	0.106
N	68	12.552	10.086	5.173
N	69	13.549	12.013	4.860
N	70	11.500	18.373	3.781
N	71	10.649	16.034	2.382
N	72	11.235	13.616	-2.876
N	73	10.493	15.585	-0.422
O	74	10.367	17.626	5.618
O	75	10.443	21.611	4.942
O	76	8.858	14.089	-1.013
O	77	8.691	17.224	2.304
S	78	12.517	13.439	1.070
S	79	13.461	14.958	4.058
S	80	14.615	19.498	3.936
S	81	16.945	17.147	-1.392
S	82	19.802	14.497	1.590
S	83	17.249	13.626	4.068
S	84	16.071	14.304	0.654
S	85	17.097	17.202	2.451
S	86	13.772	17.108	0.912

86

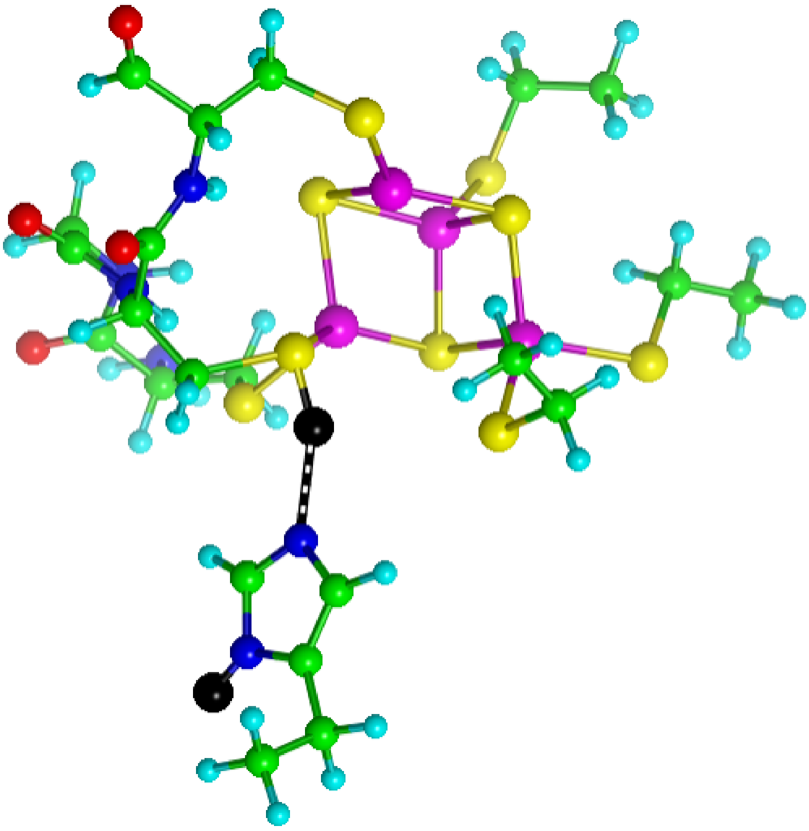

redv318nh19hpbtm_7.cmmf

S19-H^exo^

C	1	12.877	6.776	9.695
C	2	13.960	7.255	8.712
C	3	13.651	8.551	8.034
C	4	14.208	9.809	8.146
C	5	12.628	10.020	6.695
C	6	12.494	13.582	2.205
C	7	10.642	14.777	7.269
C	8	10.872	15.959	8.240
C	9	11.647	13.641	7.506
C	10	11.873	18.156	8.606
C	11	10.617	19.017	8.702
C	12	13.037	18.998	8.058
C	13	19.032	18.745	3.252
C	14	17.553	18.790	2.877
C	15	21.499	15.020	2.677
C	16	20.225	15.277	3.489
C	17	17.409	13.922	9.266
C	18	18.176	13.078	8.255
C	19	11.087	13.496	1.621
C	20	10.005	14.359	2.288
C	21	9.516	16.167	3.876
C	22	9.548	15.903	5.388
H	23	13.804	12.707	7.298
H	24	12.024	7.974	6.753
Fe	25	16.062	16.256	4.116
Fe	26	17.561	14.243	5.271
Fe	27	14.199	14.501	4.916
Fe	28	15.199	16.950	6.471
H	29	19.649	18.410	2.404
H	30	19.383	19.748	3.565
H	31	14.911	7.389	9.252
H	32	8.501	15.951	3.523
H	33	15.058	10.117	8.749
H	34	13.172	5.832	10.178
H	35	12.704	7.526	10.480
H	36	11.918	6.599	9.184
H	37	11.958	10.413	5.933
H	38	14.150	6.465	7.962
H	39	13.142	12.870	1.670
H	40	11.186	14.621	5.198
H	41	12.923	14.581	2.037
H	42	13.059	19.932	8.633
H	43	12.864	19.245	6.998
H	44	12.129	17.829	9.626
H	45	9.841	18.797	7.928
H	46	9.651	14.399	7.582
H	47	11.552	13.329	8.553
H	48	11.389	12.801	6.851
H	49	21.383	14.127	2.039
H	50	10.720	12.460	1.770
H	51	9.699	17.243	3.742
H	52	21.729	15.884	2.027
H	53	16.519	13.383	9.630
H	54	11.429	15.487	3.244
H	55	17.062	14.856	8.803
H	56	10.180	13.745	-0.201
H	57	18.043	14.176	10.135
H	58	11.761	13.293	-0.314
H	59	18.524	12.136	8.713
H	60	19.064	13.609	7.882
H	61	22.361	14.844	3.341
H	62	20.363	16.164	4.124
H	63	19.376	15.490	2.824
H	64	16.948	19.076	3.749
H	65	17.372	19.542	2.090
H	66	12.051	16.935	6.848

H	67	19.182	18.050	4.088
N	68	12.634	8.707	7.098
N	69	13.564	10.704	7.322
N	70	11.616	16.994	7.773
N	71	10.581	15.148	5.858
N	72	11.112	13.901	0.193
N	73	10.429	15.395	3.064
O	74	10.401	15.915	9.385
O	75	10.459	19.905	9.527
O	76	8.807	14.116	2.071
O	77	8.684	16.412	6.115
S	78	12.561	13.145	4.004
S	79	13.459	14.041	7.251
S	80	14.699	18.213	8.283
S	81	16.900	17.177	2.215
S	82	19.757	13.862	4.592
S	83	17.123	12.611	6.806
S	84	16.065	14.053	3.661
S	85	17.332	16.499	5.947
S	86	13.904	16.808	4.681

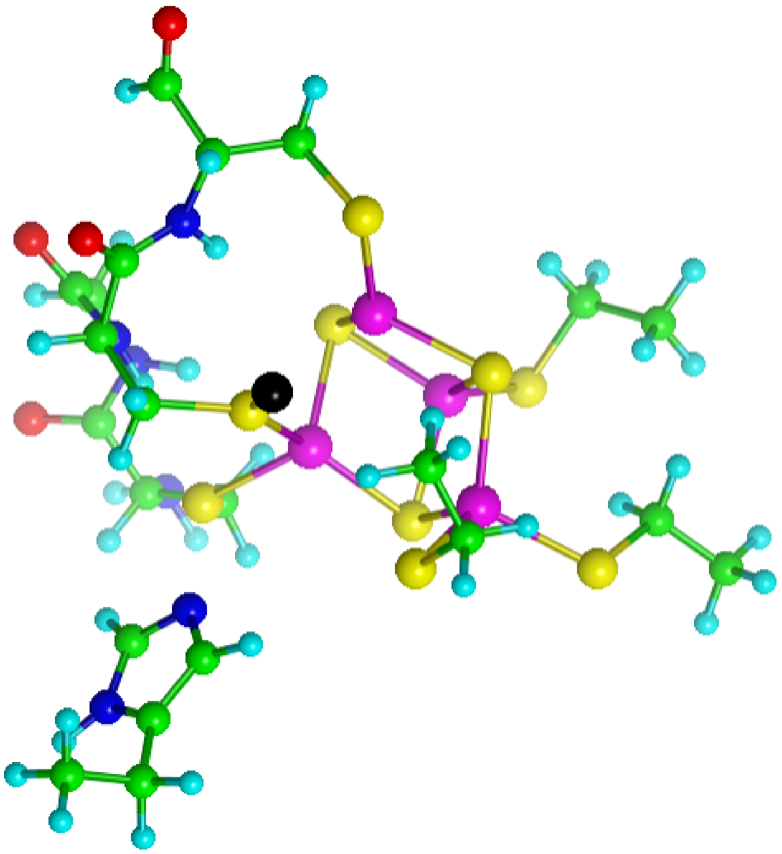

dir319httpbth_orig.cmmf

TS: S^{19}-H^{inv}

86

C	1	13.067	4.917	9.732
C	2	14.367	4.781	8.921
C	3	14.571	5.893	7.948
C	4	15.444	6.963	7.930
C	5	14.186	7.239	6.209
C	6	15.743	9.700	2.184
C	7	13.656	12.568	6.491
C	8	14.010	13.901	7.182
C	9	14.391	11.401	7.174
C	10	15.609	15.748	7.347
C	11	14.780	16.980	7.002
C	12	17.087	15.972	6.966
C	13	23.371	12.809	3.732
C	14	22.085	13.168	2.993
C	15	24.549	8.520	4.437
C	16	23.353	9.363	4.893
C	17	19.382	10.300	10.048
C	18	20.026	9.061	9.434
C	19	14.461	9.839	1.366
C	20	13.605	11.086	1.634
C	21	13.480	13.286	2.724
C	22	13.200	13.400	4.230
H	23	16.649	11.584	8.129
Fe	24	19.676	11.525	4.295
Fe	25	20.240	9.590	6.207
Fe	26	17.294	10.634	4.900
Fe	27	18.425	12.985	6.122
H	28	23.973	12.094	3.151
H	29	23.979	13.714	3.916
H	30	15.229	4.772	9.605
H	31	12.511	13.280	2.210
H	32	16.272	7.174	8.604
H	33	12.959	4.095	10.461
H	34	13.056	5.871	10.280
H	35	12.180	4.903	9.079
H	36	13.767	7.611	5.276
H	37	14.378	3.802	8.406
H	38	16.220	8.741	1.924
H	39	14.375	11.731	4.642
H	40	16.466	10.488	1.920
H	41	17.393	16.933	7.400
H	42	17.191	16.044	5.872
H	43	15.545	15.612	8.439
H	44	14.102	16.855	6.121

H	45	12.577	12.448	6.702
H	46	14.240	11.468	8.258
H	47	13.994	10.440	6.825
H	48	24.212	7.568	3.999
H	49	13.791	8.996	1.632
H	50	14.013	14.206	2.439
H	51	25.136	9.066	3.676
H	52	18.321	10.112	10.279
H	53	15.161	11.985	2.629
H	54	19.431	11.144	9.343
H	55	13.916	9.837	-0.610
H	56	19.893	10.603	10.980
H	57	15.317	9.020	-0.315
H	58	19.988	8.215	10.142
H	59	21.083	9.246	9.191
H	60	25.212	8.283	5.285
H	61	23.713	10.305	5.329
H	62	22.720	9.631	4.036
H	63	21.467	13.836	3.610
H	64	22.308	13.700	2.054
H	65	15.655	14.114	5.974
H	66	23.132	12.353	4.702
H	67	13.013	5.478	6.526
N	68	13.764	6.087	6.832
N	69	15.197	7.783	6.853
N	70	15.075	14.574	6.679
N	71	13.878	12.546	5.049
N	72	14.791	9.866	-0.080
N	73	14.212	12.122	2.278
O	74	13.377	14.269	8.180
O	75	14.846	18.036	7.613
O	76	12.432	11.124	1.230
O	77	12.434	14.280	4.643
S	78	15.422	9.671	4.009
S	79	16.233	11.270	6.881
S	80	18.219	14.672	7.638
S	81	21.078	11.684	2.521
S	82	22.300	8.521	6.165
S	83	19.146	8.511	7.902
S	84	19.099	9.336	4.328
S	85	20.464	11.944	6.338
S	86	17.745	12.759	4.027

86

C	1	7.559	11.661	9.826
C	2	8.773	12.243	9.081
C	3	8.391	13.238	8.036
C	4	8.454	14.616	7.993
C	5	7.458	14.042	6.175
C	6	7.785	16.921	1.975
C	7	4.241	18.189	6.026
C	8	3.845	19.524	6.691
C	9	5.374	17.513	6.806
C	10	4.136	21.957	6.715
C	11	2.803	22.479	6.183
C	12	5.272	22.950	6.418
C	13	12.362	23.624	3.641
C	14	11.183	23.208	2.766
C	15	15.619	20.706	4.719
C	16	14.131	20.765	5.071
C	17	9.911	19.623	9.970
C	18	11.137	18.919	9.400
C	19	6.691	16.249	1.151
C	20	5.282	16.849	1.257
C	21	3.896	18.629	2.233
C	22	3.536	18.610	3.726

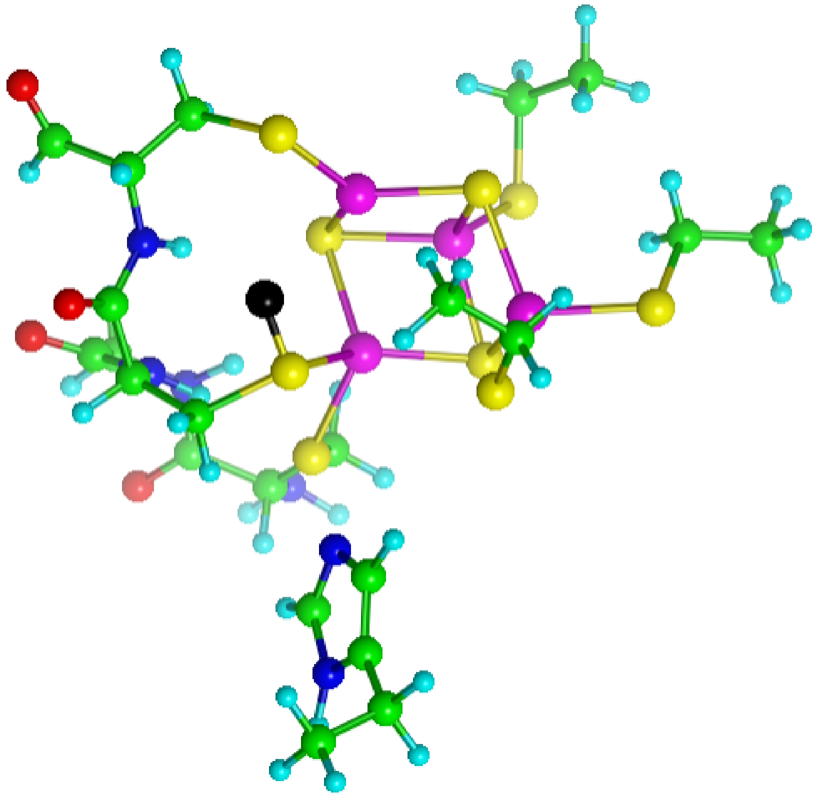

dir319httpbtb_3.cmmf

S19-Hendo

H	23	6.584	19.600	7.149
Fe	24	9.976	20.685	4.188
Fe	25	11.321	19.364	6.193
Fe	26	8.329	18.665	4.793
Fe	27	8.261	21.501	5.969
H	28	13.317	23.333	3.176
H	29	12.367	24.720	3.795
H	30	9.437	12.750	9.798
H	31	3.123	18.065	1.696
H	32	8.920	15.296	8.704
H	33	7.868	10.946	10.607
H	34	6.980	12.467	10.300
H	35	6.883	11.133	9.136
H	36	6.980	14.067	5.198
H	37	9.369	11.420	8.644
H	38	8.738	16.398	1.792
H	39	5.424	17.874	4.265
H	40	7.941	17.965	1.664
H	41	4.916	23.944	6.721
H	42	5.487	22.978	5.340
H	43	4.023	21.869	7.808
H	44	2.372	21.895	5.330
H	45	3.340	17.570	6.171
H	46	5.029	17.403	7.843
H	47	5.595	16.517	6.401
H	48	15.882	19.720	4.304
H	49	6.566	15.220	1.551
H	50	3.833	19.682	1.915
H	51	15.875	21.477	3.970
H	52	9.103	18.902	10.168
H	53	6.013	18.443	2.306
H	54	9.522	20.363	9.256
H	55	6.301	15.765	-0.792
H	56	10.152	20.145	10.915
H	57	7.906	15.737	-0.420
H	58	11.541	18.196	10.129
H	59	11.938	19.634	9.161
H	60	16.244	20.867	5.613
H	61	13.882	21.748	5.496
H	62	13.516	20.641	4.168
H	63	10.233	23.471	3.255
H	64	11.208	23.735	1.797
H	65	5.084	20.550	5.406
H	66	12.292	23.141	4.624
H	67	7.545	11.938	6.548
N	68	7.741	12.884	6.859
N	69	7.870	15.099	6.843
N	70	4.417	20.642	6.174
N	71	4.529	18.285	4.601
N	72	7.040	16.265	-0.288
N	73	5.175	18.064	1.863
O	74	3.093	19.533	7.672
O	75	2.235	23.460	6.638
O	76	4.316	16.238	0.772
O	77	2.395	18.943	4.076
S	78	7.425	16.801	3.791
S	79	7.042	18.334	6.883
S	80	6.824	22.585	7.352
S	81	11.152	21.393	2.381
S	82	13.635	19.474	6.304
S	83	10.738	17.954	7.874
S	84	10.551	18.510	4.318
S	85	10.495	21.581	6.174
S	86	7.681	20.755	3.982

86

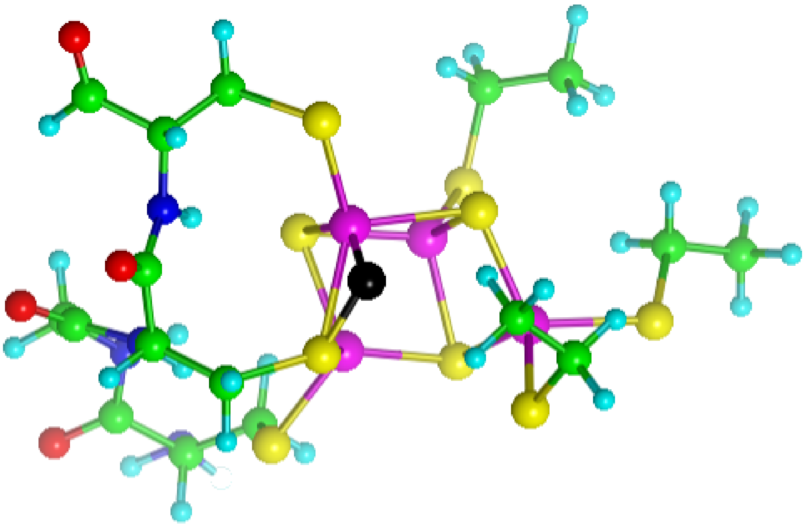

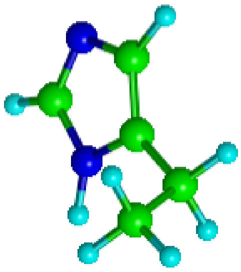

redv3nh19h4pbth_1_8777.43.cmmf

TS: S19-Fe4

C	1	11.079	10.043	9.234
C	2	11.905	10.676	8.097
C	3	11.119	11.580	7.203
C	4	11.033	12.956	7.109
C	5	9.643	12.211	5.653
C	6	9.440	16.068	0.656
C	7	7.519	17.492	5.701
C	8	7.350	18.858	6.398
C	9	8.838	16.816	6.101
C	10	7.726	21.278	6.212
C	11	6.376	21.971	6.053
C	12	8.853	22.097	5.551
C	13	14.934	22.344	1.395
C	14	13.483	22.136	0.970
C	15	18.089	19.132	1.288
C	16	16.791	19.283	2.091
C	17	14.044	17.977	7.894
C	18	15.071	17.253	7.025
C	19	8.098	15.548	0.143
C	20	6.838	16.217	0.714
C	21	5.952	17.942	2.227
C	22	6.096	17.871	3.755
H	23	11.344	18.656	6.104
Fe	24	12.445	19.413	2.526
Fe	25	14.271	17.848	3.892
Fe	26	10.941	17.381	3.235
Fe	27	11.047	19.628	4.853
H	28	15.625	21.974	0.623
H	29	15.143	23.415	1.576
H	30	12.728	11.266	8.528
H	31	5.019	17.433	1.955
H	32	11.610	13.701	7.654
H	33	11.702	9.382	9.859
H	34	10.650	10.824	9.878
H	35	10.244	9.443	8.838
H	36	8.921	12.131	4.843
H	37	12.384	9.877	7.504
H	38	10.251	15.558	0.111
H	39	8.033	17.086	3.653
H	40	9.563	17.142	0.449
H	41	8.756	23.139	5.883
H	42	8.745	22.070	4.452
H	43	7.925	21.214	7.294
H	44	5.654	21.448	5.374
H	45	6.714	16.887	6.157
H	46	8.909	16.836	7.197
H	47	8.837	15.761	5.786
H	48	18.083	18.193	0.714
H	49	8.003	14.497	0.482
H	50	5.836	19.011	1.984
H	51	18.202	19.974	0.581
H	52	13.299	17.270	8.290
H	53	7.969	17.674	1.610
H	54	13.509	18.733	7.301
H	55	7.104	15.297	-1.620
H	56	14.532	18.487	8.745
H	57	8.732	15.094	-1.755
H	58	15.614	16.500	7.619
H	59	15.814	17.960	6.622
H	60	18.968	19.115	1.953
H	61	16.812	20.232	2.648
H	62	15.923	19.318	1.419
H	63	12.797	22.487	1.754
H	64	13.260	22.712	0.055
H	65	8.194	19.778	4.776
H	66	15.135	21.794	2.324

H	67	10.055	10.144	6.013
N	68	10.211	11.116	6.257
N	69	10.114	13.334	6.154
N	70	7.698	19.941	5.655
N	71	7.316	17.528	4.256
N	72	8.010	15.680	-1.330
N	73	7.015	17.352	1.445
O	74	6.947	18.918	7.565
O	75	6.082	23.021	6.605
O	76	5.724	15.717	0.485
O	77	5.136	18.202	4.462
S	78	9.651	15.703	2.467
S	79	10.545	17.325	5.447
S	80	10.535	21.511	6.028
S	81	13.098	20.367	0.567
S	82	16.530	17.925	3.326
S	83	14.298	16.338	5.610
S	84	12.963	17.197	2.208
S	85	13.352	19.907	4.496
S	86	10.162	19.516	2.800

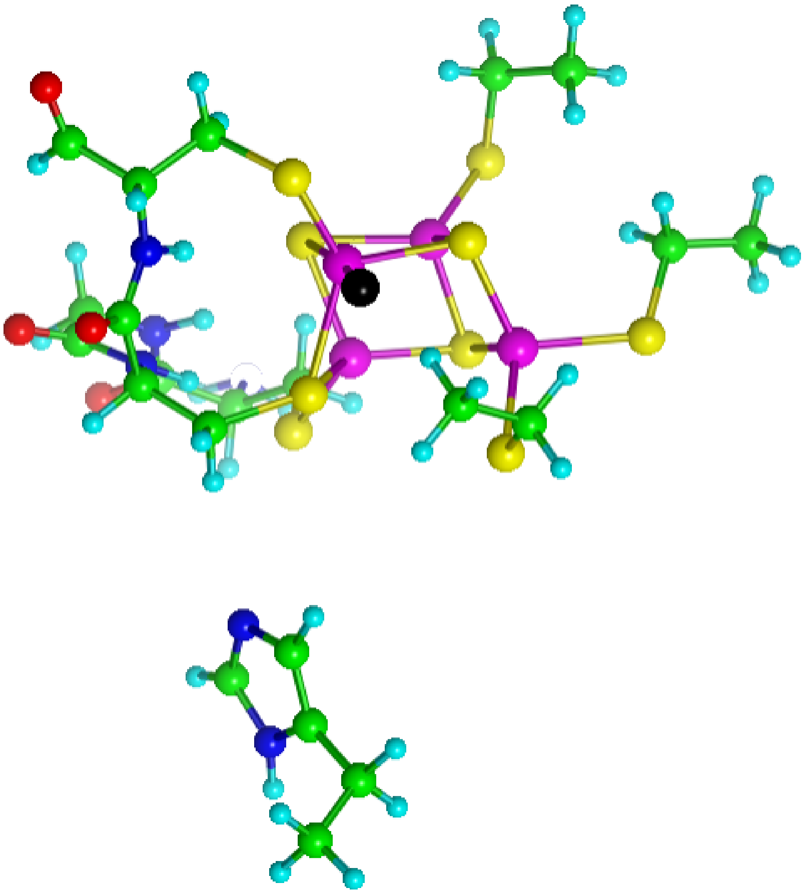

redv318nh19hdpb_5.cmmf

Fe4-H

86

C	1	8.954	6.910	13.609
C	2	9.728	7.989	12.833
C	3	8.858	8.981	12.134
C	4	8.526	10.291	12.415
C	5	7.434	9.812	10.629
C	6	8.668	15.100	7.051
C	7	5.804	15.097	11.808
C	8	5.497	16.294	12.732
C	9	7.056	14.324	12.254
C	10	5.788	18.713	13.057
C	11	4.516	19.474	12.711
C	12	7.034	19.608	12.875
C	13	13.515	20.766	10.501
C	14	12.180	20.681	9.772
C	15	16.754	17.661	10.115
C	16	15.317	17.611	10.633
C	17	11.560	14.758	15.208
C	18	12.789	14.420	14.366
C	19	7.488	14.715	6.160
C	20	6.103	15.226	6.585
C	21	4.817	16.384	8.333
C	22	4.696	15.928	9.792
H	23	9.744	16.852	13.740
Fe	24	11.021	17.709	10.229
Fe	25	12.577	15.777	11.407
Fe	26	9.576	15.595	10.170
Fe	27	9.454	17.262	12.304
H	28	14.356	20.665	9.798
H	29	13.613	21.733	11.030
H	30	10.376	8.548	13.525
H	31	4.000	15.929	7.758
H	32	8.909	10.917	13.218
H	33	9.640	6.207	14.106
H	34	8.313	7.371	14.375
H	35	8.300	6.326	12.942
H	36	6.811	9.861	9.740
H	37	10.411	7.506	12.110
H	38	9.601	14.765	6.575
H	39	6.646	15.163	9.836
H	40	8.754	16.190	7.167
H	41	6.841	20.555	13.400
H	42	7.198	19.822	11.807
H	43	5.715	18.423	14.118
H	44	3.961	19.078	11.824

H	45	4.955	14.418	11.994
H	46	6.904	14.094	13.318
H	47	7.105	13.367	11.714
H	48	16.904	16.927	9.308
H	49	7.378	13.612	6.226
H	50	4.650	17.474	8.342
H	51	16.993	18.666	9.721
H	52	10.874	13.899	15.267
H	53	6.931	16.282	8.133
H	54	11.002	15.592	14.762
H	55	6.845	14.957	4.251
H	56	11.860	15.043	16.234
H	57	8.459	14.640	4.353
H	58	13.355	13.590	14.820
H	59	13.469	15.281	14.298
H	60	17.468	17.424	10.920
H	61	15.179	18.366	11.421
H	62	14.605	17.848	9.829
H	63	11.346	20.749	10.484
H	64	12.069	21.511	9.056
H	65	6.562	17.513	11.485
H	66	13.590	19.961	11.243
H	67	8.190	7.821	10.455
N	68	8.139	8.686	10.981
N	69	7.640	10.792	11.484
N	70	5.902	17.506	12.267
N	71	5.821	15.445	10.391
N	72	7.699	15.181	4.772
N	73	6.055	16.057	7.659
O	74	4.922	16.120	13.812
O	75	4.109	20.445	13.334
O	76	5.098	14.874	5.945
O	77	3.619	16.092	10.379
S	78	8.561	14.259	8.704
S	79	8.828	14.938	12.185
S	80	8.532	18.839	13.617
S	81	11.999	19.125	8.784
S	82	14.884	15.963	11.347
S	83	12.376	13.876	12.645
S	84	11.679	15.735	9.373
S	85	11.672	17.684	12.341
S	86	8.749	17.697	10.150

86				
C	1	10.249	5.696	13.054
C	2	11.417	6.353	12.296
C	3	11.073	7.653	11.645
C	4	11.407	8.959	11.940
C	5	10.113	9.092	10.234
C	6	10.779	13.755	6.476
C	7	8.081	14.086	11.322
C	8	7.896	15.159	12.409
C	9	9.265	13.162	11.654
C	10	8.553	17.398	13.173
C	11	7.279	18.234	13.166
C	12	9.787	18.293	12.936
C	13	16.640	18.839	9.607
C	14	15.233	18.913	9.020
C	15	19.438	15.365	9.002
C	16	18.037	15.492	9.613
C	17	14.272	13.074	14.424
C	18	15.367	12.462	13.555
C	19	9.508	13.618	5.640
C	20	8.239	14.289	6.193
C	21	7.285	15.741	7.939
C	22	7.109	15.253	9.388

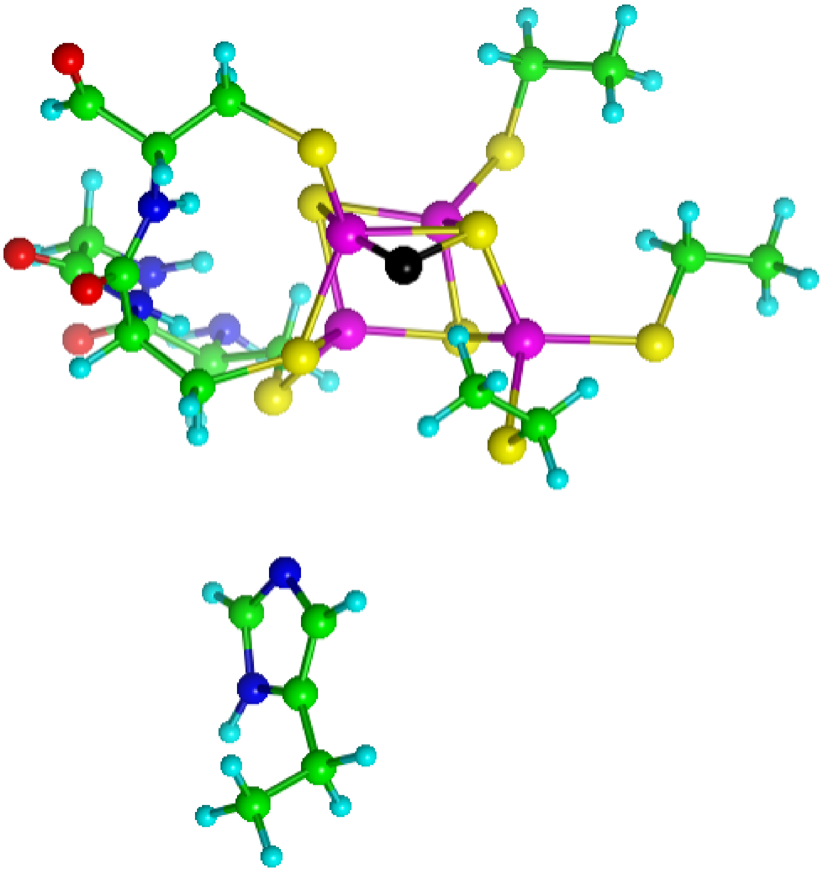

redv3423pbtj_1_8779.17.cmmf

TS: Fe4-S3

H	23	12.948	15.312	12.697
Fe	24	13.641	16.092	9.547
Fe	25	15.116	14.009	10.624
Fe	26	11.895	14.173	9.566
Fe	27	11.914	15.900	11.671
H	28	17.381	18.607	8.827
H	29	16.922	19.794	10.090
H	30	12.242	6.547	12.997
H	31	6.370	15.492	7.390
H	32	12.075	9.320	12.718
H	33	10.565	4.754	13.528
H	34	9.868	6.369	13.836
H	35	9.409	5.467	12.380
H	36	9.530	9.458	9.391
H	37	11.815	5.642	11.550
H	38	11.603	13.249	5.947
H	39	8.928	14.240	9.365
H	40	11.080	14.806	6.582
H	41	9.726	19.138	13.635
H	42	9.777	18.691	11.909
H	43	8.639	16.941	14.171
H	44	6.649	18.113	12.250
H	45	7.168	13.474	11.418
H	46	9.098	12.785	12.672
H	47	9.257	12.297	10.977
H	48	19.455	14.588	8.221
H	49	9.238	12.544	5.610
H	50	7.359	16.839	7.993
H	51	19.744	16.324	8.547
H	52	13.384	12.426	14.460
H	53	9.344	15.368	7.546
H	54	13.950	14.041	14.016
H	55	8.871	14.013	3.738
H	56	14.633	13.238	15.456
H	57	10.474	13.659	3.825
H	58	15.699	11.500	13.979
H	59	16.247	13.118	13.502
H	60	20.183	15.090	9.766
H	61	18.041	16.286	10.372
H	62	17.304	15.777	8.846
H	63	14.491	19.104	9.806
H	64	15.160	19.739	8.291
H	65	9.113	16.423	11.360
H	66	16.686	18.046	10.365
H	67	9.812	6.984	10.035
N	68	10.232	7.756	10.540
N	69	10.804	9.839	11.068
N	70	8.489	16.357	12.166
N	71	8.163	14.604	9.957
N	72	9.722	14.182	4.282
N	73	8.402	15.198	7.193
O	74	7.281	14.900	13.450
O	75	6.959	18.999	14.065
O	76	7.134	13.998	5.705
O	77	6.061	15.529	9.988
S	78	10.619	12.965	8.152
S	79	11.073	13.698	11.634
S	80	11.386	17.424	13.255
S	81	14.773	17.368	8.101
S	82	17.436	13.944	10.436
S	83	14.812	12.099	11.826
S	84	13.995	14.023	8.699
S	85	14.309	15.941	11.676
S	86	11.395	16.418	9.514

86

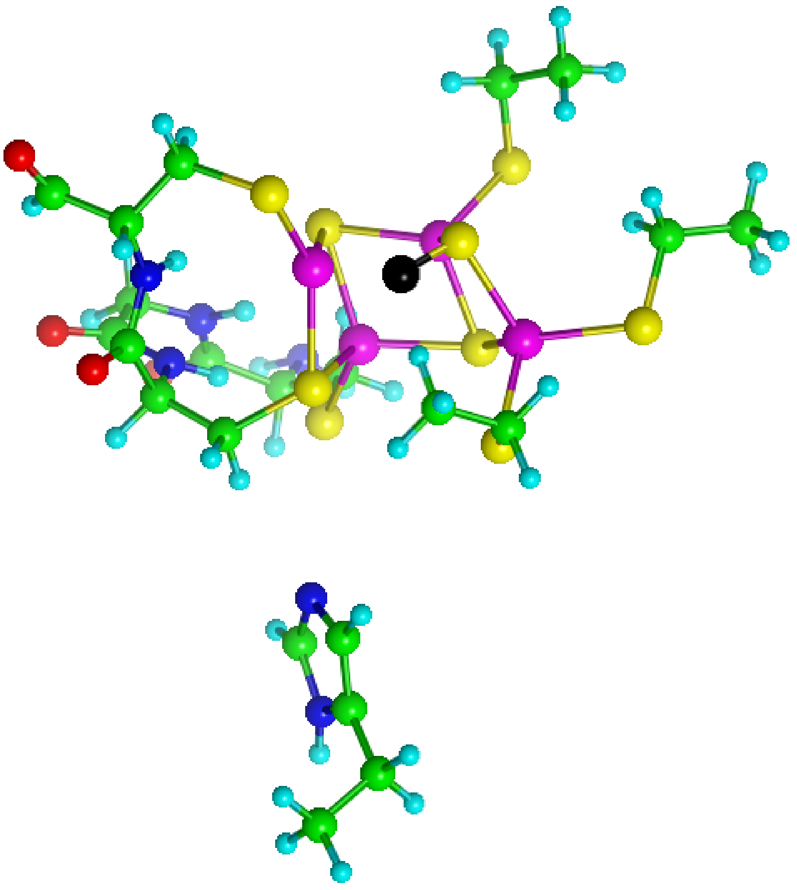

redv318nh19hepb_4.cmmf

S3-H

C	1	6.242	6.380	15.120
C	2	7.345	7.336	14.632
C	3	6.826	8.547	13.929
C	4	6.692	9.867	14.315
C	5	5.868	9.783	12.336
C	6	6.654	14.858	8.858
C	7	3.225	14.420	13.215
C	8	2.662	15.370	14.284
C	9	4.481	13.704	13.738
C	10	2.619	17.672	15.143
C	11	1.241	18.247	14.840
C	12	3.665	18.800	15.219
C	13	10.882	20.553	13.539
C	14	9.715	20.329	12.583
C	15	14.274	17.592	13.480
C	16	12.798	17.445	13.835
C	17	8.511	14.206	17.563
C	18	9.836	13.822	16.912
C	19	5.559	14.570	7.833
C	20	4.158	15.111	8.151
C	21	2.735	16.195	9.837
C	22	2.403	15.572	11.202
H	23	7.790	16.711	15.540
Fe	24	8.536	17.340	12.743
Fe	25	10.114	15.484	14.128
Fe	26	7.019	15.412	12.078
Fe	27	6.116	16.852	14.165
H	28	11.841	20.520	13.000
H	29	10.794	21.534	14.042
H	30	7.940	7.678	15.492
H	31	1.973	15.863	9.123
H	32	7.026	10.328	15.242
H	33	6.666	5.501	15.630
H	34	5.567	6.897	15.818
H	35	5.627	6.019	14.280
H	36	5.433	10.044	11.374
H	37	8.047	6.784	13.981
H	38	7.619	14.508	8.460
H	39	4.331	14.807	11.431
H	40	6.765	15.939	9.029
H	41	3.298	19.540	15.944
H	42	3.755	19.296	14.240
H	43	2.563	17.169	16.121
H	44	0.858	18.012	13.815
H	45	2.442	13.648	13.123
H	46	4.218	13.275	14.713
H	47	4.749	12.870	13.076
H	48	14.547	16.910	12.659
H	49	5.409	13.469	7.819
H	50	2.627	17.286	9.959
H	51	14.495	18.626	13.159
H	52	7.736	13.455	17.348
H	53	4.854	16.008	9.855
H	54	8.148	15.167	17.174
H	55	5.140	14.945	5.878
H	56	8.618	14.301	18.660
H	57	6.723	14.575	6.144
H	58	10.226	12.891	17.356
H	59	10.596	14.604	17.063
H	60	14.917	17.352	14.342
H	61	12.534	18.147	14.641
H	62	12.165	17.692	12.971
H	63	8.763	20.315	13.133
H	64	9.653	21.144	11.844
H	65	3.562	16.965	13.356
H	66	10.905	19.775	14.314

H	67	6.256	7.702	12.036
N	68	6.285	8.511	12.648
N	69	6.095	10.618	13.328
N	70	2.990	16.683	14.152
N	71	3.441	15.040	11.909
N	72	5.932	15.111	6.507
N	73	4.026	15.868	9.273
O	74	2.005	14.924	15.231
O	75	0.594	18.928	15.622
O	76	3.211	14.839	7.393
O	77	1.245	15.650	11.632
S	78	6.355	13.952	10.451
S	79	6.114	14.579	14.019
S	80	5.325	18.231	15.781
S	81	9.883	18.757	11.618
S	82	12.378	15.738	14.412
S	83	9.656	13.505	15.100
S	84	9.303	15.338	12.104
S	85	8.916	17.351	15.055
S	86	6.359	17.595	12.085

Table S3. Pictures and coordinates for some other relevant calculated structures

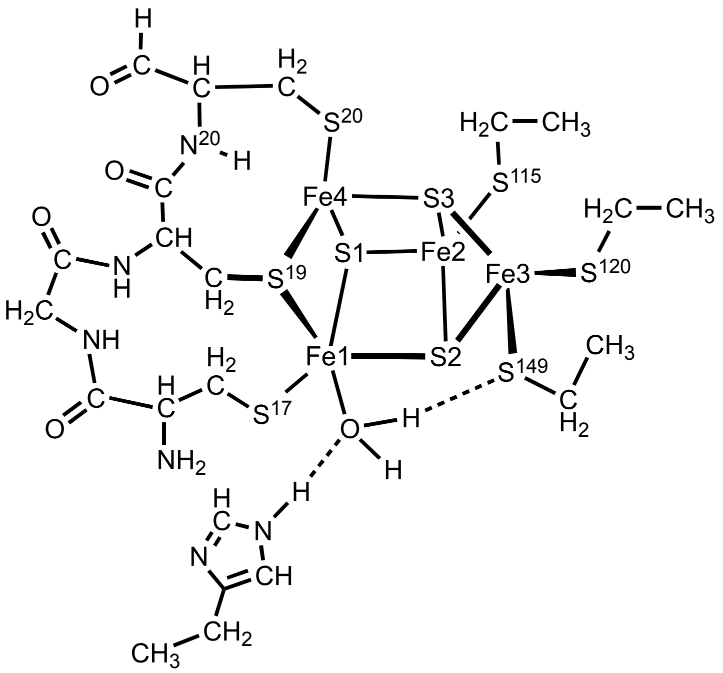

88				
C	1	12.068	9.077	9.188
C	2	13.202	8.985	8.140
C	3	12.977	9.834	6.916
C	4	13.790	10.830	6.398
C	5	12.013	10.550	5.115
C	6	12.600	13.499	-0.482
C	7	10.416	16.282	3.928
C	8	10.482	17.725	4.485
C	9	11.485	15.374	4.586
C	10	11.658	19.889	4.399
C	11	10.500	20.862	4.205
C	12	12.936	20.449	3.711
C	13	19.312	18.739	-0.298
C	14	17.821	18.875	-0.644
C	15	21.700	14.964	0.273
C	16	20.505	15.532	1.046
C	17	17.159	15.635	6.618
C	18	18.119	14.523	6.182
C	19	11.258	13.344	-1.211
C	20	10.184	14.424	-0.948
C	21	9.650	16.586	0.140
C	22	9.491	16.730	1.674
H	23	15.488	13.260	4.129
H	24	15.143	12.493	2.813
H	25	13.574	11.967	4.584
Fe	26	16.061	16.421	0.985
Fe	27	17.671	14.842	2.795
Fe	28	14.137	14.729	2.282
Fe	29	14.621	17.381	3.188
H	30	19.848	18.184	-1.082
H	31	19.783	19.736	-0.190
H	32	14.160	9.274	8.601
H	33	8.665	16.344	-0.266
H	34	14.732	11.260	6.723
H	35	12.242	8.381	10.025
H	36	11.992	10.096	9.593
H	37	11.106	8.831	8.720
H	38	11.332	10.707	4.286
H	39	13.310	7.929	7.834
H	40	13.281	12.709	-0.830
H	41	11.139	15.522	2.058

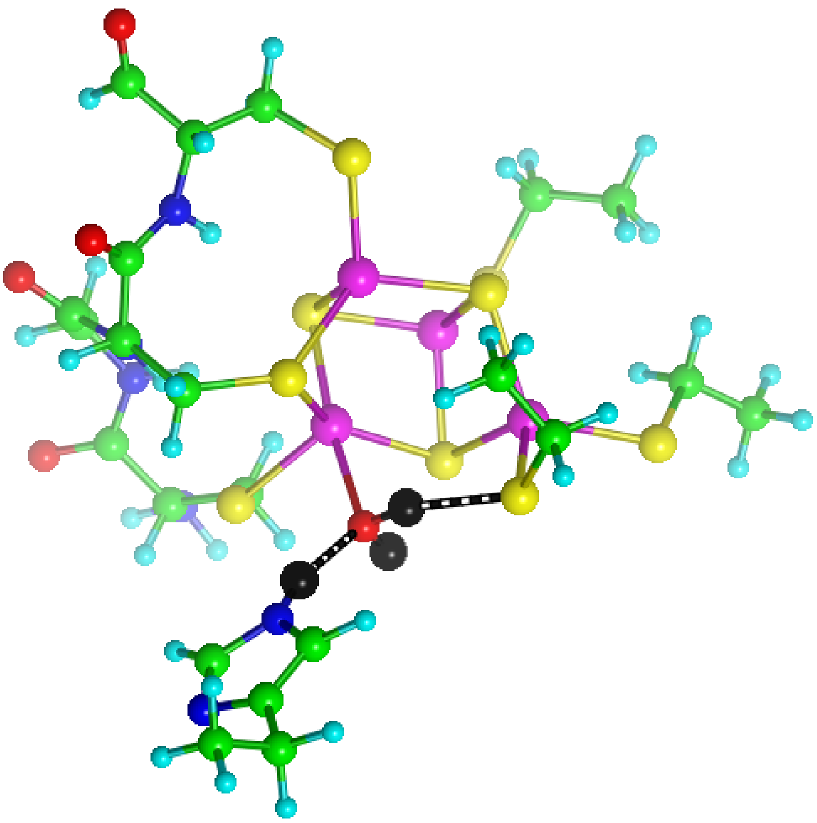

redv318n19oh2b_5.cmmf

H	42	13.086	14.451	-0.724
H	43	13.073	21.478	4.064
H	44	12.790	20.475	2.621
H	45	11.851	19.807	5.477
H	46	9.795	20.588	3.383
H	47	9.436	15.918	4.270
H	48	11.334	15.442	5.670
H	49	11.290	14.342	4.275
H	50	21.451	13.980	-0.153
H	51	10.785	12.416	-0.843
H	52	9.941	17.580	-0.233
H	53	22.002	15.634	-0.555
H	54	16.185	15.227	6.922
H	55	11.570	15.684	-0.078
H	56	16.981	16.336	5.795
H	57	10.557	13.100	-3.129
H	58	17.579	16.201	7.471
H	59	12.087	12.526	-2.917
H	60	18.312	13.828	7.015
H	61	19.083	14.947	5.869
H	62	22.569	14.825	0.938
H	63	20.763	16.516	1.460
H	64	19.654	15.671	0.372
H	65	17.284	19.355	0.183
H	66	17.691	19.503	-1.540
H	67	11.978	18.178	3.162
H	68	19.433	18.198	0.646
N	69	11.860	9.664	6.103
N	70	13.162	11.280	5.251
N	71	11.325	18.578	3.839
N	72	10.462	16.185	2.460
N	73	11.463	13.309	-2.699
N	74	10.588	15.579	-0.342
O	75	9.827	18.041	5.493
O	76	10.352	21.901	4.845
O	77	14.707	12.944	3.567
O	78	9.008	14.217	-1.312
O	79	8.531	17.391	2.111
S	80	12.433	13.320	1.376
S	81	13.361	15.595	4.356
S	82	14.499	19.512	4.131
S	83	16.996	17.234	-1.021
S	84	19.987	14.414	2.449
S	85	17.520	13.479	4.743
S	86	16.176	14.127	1.109
S	87	16.961	17.127	2.955
S	88	13.774	16.754	1.150

88

C	1	12.147	5.501	9.507
C	2	13.180	4.603	8.782
C	3	13.740	5.304	7.575
C	4	14.800	6.191	7.534
C	5	13.755	6.181	5.596
C	6	13.598	12.102	2.410
C	7	12.786	12.879	8.011
C	8	13.105	13.932	9.102
C	9	13.840	11.741	7.983
C	10	14.389	15.969	9.666
C	11	13.316	16.975	10.067
C	12	15.592	16.732	9.034
C	13	21.360	15.906	3.845
C	14	19.968	16.371	3.391
C	15	23.311	11.981	2.817
C	16	21.924	12.249	3.399
C	17	19.398	10.777	9.495

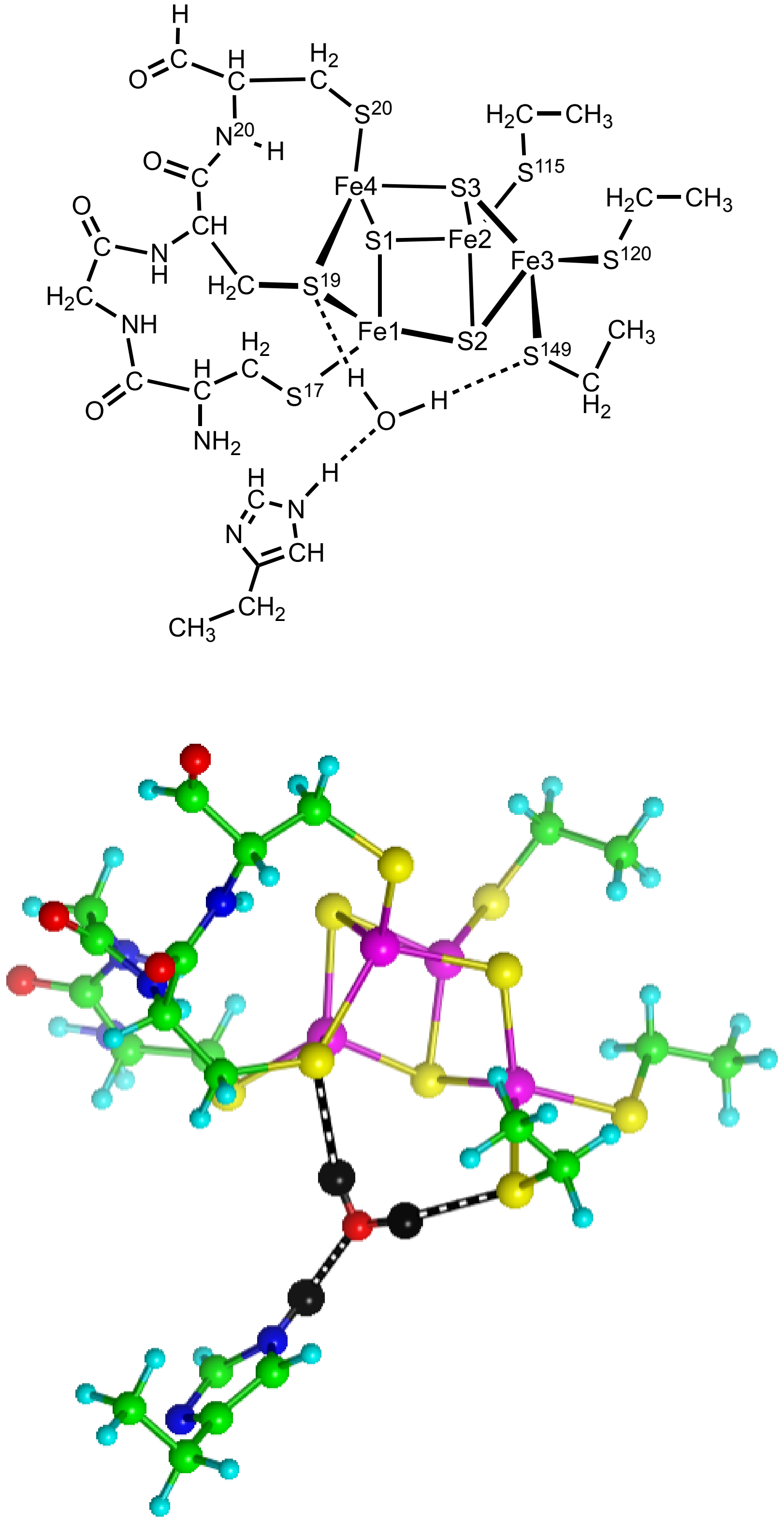

redv318noh2bb_7.cmmf

C	18	20.261	10.023	8.484
C	19	12.088	12.235	2.154
C	20	11.323	13.316	2.953
C	21	11.408	14.809	4.937
C	22	11.529	14.281	6.387
H	23	17.286	9.015	6.568
H	24	16.019	9.810	6.184
H	25	15.422	7.561	6.024
Fe	26	18.046	13.901	4.550
Fe	27	19.376	11.539	5.551
Fe	28	15.824	12.468	5.025
Fe	29	16.832	14.156	7.037
H	30	21.269	15.182	4.662
H	31	21.963	16.760	4.211
H	32	13.995	4.342	9.477
H	33	10.344	14.882	4.700
H	34	15.525	6.503	8.275
H	35	11.706	4.991	10.380
H	36	12.618	6.433	9.845
H	37	11.332	5.767	8.820
H	38	13.505	6.459	4.577
H	39	12.698	3.661	8.478
H	40	14.018	11.395	1.683
H	41	13.082	12.986	5.897
H	42	14.112	13.059	2.265
H	43	15.871	17.533	9.730
H	44	15.277	17.192	8.085
H	45	14.736	15.453	10.570
H	46	12.490	17.087	9.325
H	47	11.846	12.426	8.360
H	48	13.940	11.377	9.012
H	49	13.448	10.925	7.365
H	50	23.361	10.972	2.377
H	51	11.621	11.290	2.493
H	52	11.834	15.824	4.940
H	53	23.570	12.713	2.029
H	54	18.517	10.184	9.782
H	55	13.010	13.727	4.030
H	56	19.036	11.722	9.072
H	57	10.800	12.640	0.619
H	58	19.976	11.006	10.410
H	59	12.052	11.657	0.175
H	60	20.622	9.073	8.910
H	61	21.138	10.619	8.200
H	62	24.082	12.035	3.603
H	63	21.888	13.246	3.840
H	64	21.158	12.223	2.614
H	65	19.426	16.820	4.233
H	66	20.060	17.142	2.608
H	67	21.901	15.430	3.016
H	68	14.275	14.970	7.777
N	69	13.090	5.294	6.345
N	70	14.804	6.742	6.268
N	71	12.567	13.440	6.664
N	72	13.856	14.998	8.709
N	73	11.813	12.499	0.705
N	74	12.036	14.006	3.890
O	75	12.712	13.762	10.269
O	76	13.352	17.666	11.085
O	77	16.395	8.903	6.139
O	78	10.109	13.508	2.730
O	79	10.732	14.706	7.241
S	80	13.951	11.414	4.131
S	81	15.616	12.007	7.350
S	82	17.113	15.677	8.755
S	83	18.924	14.997	2.661

S	84	21.509	10.992	4.704
S	85	19.372	9.582	6.899
S	86	17.756	11.653	3.990
S	87	19.148	13.690	6.532
S	88	15.940	14.811	5.048

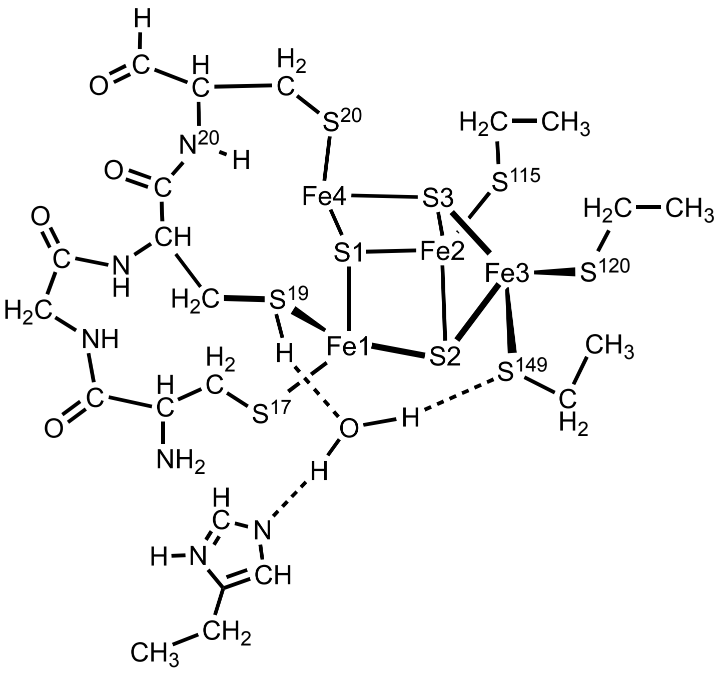

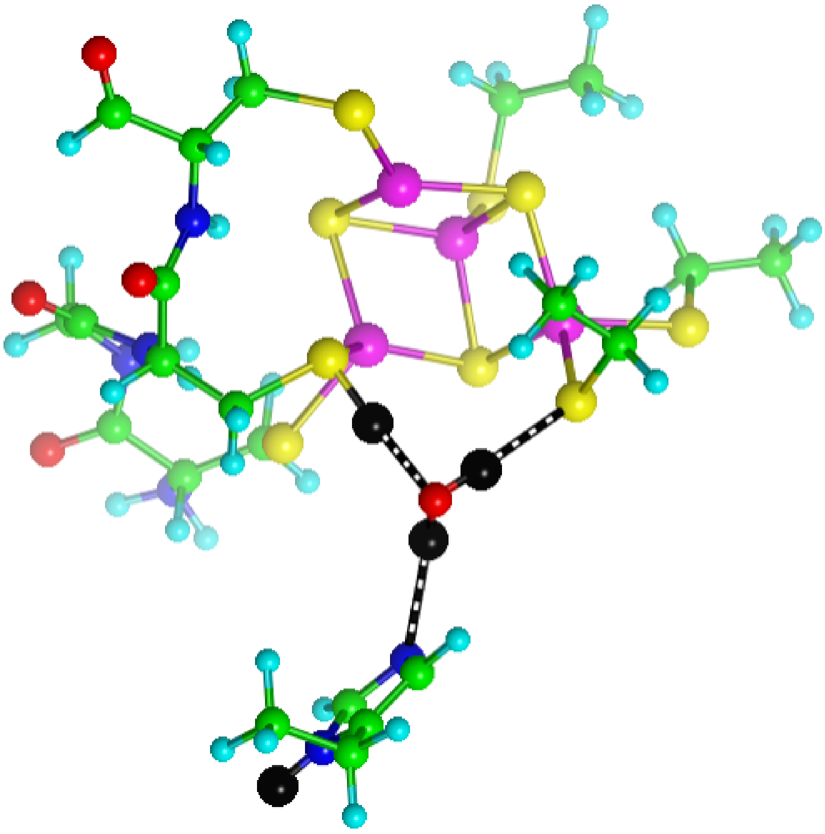

sepv318nhoh2epb_6.cmmf

89

C	1	8.816	11.630	7.433
C	2	10.166	11.144	6.881
C	3	10.435	11.602	5.486
C	4	11.330	12.534	5.010
C	5	10.160	11.965	3.301
C	6	8.683	16.498	-1.034
C	7	6.925	17.959	3.973
C	8	6.934	19.285	4.761
C	9	8.184	17.121	4.243
C	10	7.507	21.657	4.775
C	11	6.098	22.247	4.883
C	12	8.443	22.627	4.031
C	13	14.279	22.785	-0.988
C	14	12.887	22.471	-1.527
C	15	17.526	19.666	-0.989
C	16	16.097	19.492	-0.478
C	17	13.798	18.918	5.858
C	18	14.846	18.247	4.981
C	19	7.292	15.938	-1.340
C	20	6.089	16.783	-0.892
C	21	5.356	18.650	0.536
C	22	5.517	18.598	2.065
H	23	12.449	16.047	4.343
H	24	10.568	16.865	4.142
H	25	8.921	10.578	4.343
H	26	11.540	14.846	3.911
Fe	27	12.023	20.057	0.379
Fe	28	13.854	18.603	1.824
Fe	29	10.425	18.206	1.418
Fe	30	11.006	20.986	2.598
H	31	14.392	22.384	0.028
H	32	14.446	23.878	-0.950
H	33	10.976	11.554	7.505
H	34	4.379	18.213	0.291
H	35	12.054	13.114	5.578
H	36	8.698	11.362	8.494
H	37	8.738	12.723	7.341
H	38	7.968	11.192	6.883
H	39	9.726	11.895	2.308
H	40	10.240	10.044	6.967
H	41	9.436	15.852	-1.510
H	42	7.373	17.644	1.899
H	43	8.815	17.502	-1.465
H	44	8.242	23.625	4.441
H	45	8.198	22.649	2.957
H	46	7.881	21.528	5.804
H	47	5.310	21.682	4.322
H	48	6.072	17.414	4.411
H	49	8.224	16.916	5.321
H	50	8.125	16.176	3.684
H	51	17.994	18.689	-1.185
H	52	7.182	15.013	-0.734
H	53	5.330	19.717	0.256
H	54	17.540	20.254	-1.924
H	55	13.144	18.167	6.327
H	56	7.345	18.130	-0.021
H	57	13.161	19.583	5.259
H	58	6.140	15.444	-2.937
H	59	14.269	19.520	6.656

	H	60	7.681	14.879	-3.053
	H	61	15.481	17.574	5.580
	H	62	15.503	18.989	4.505
	H	63	18.146	20.187	-0.242
	H	64	15.636	20.469	-0.296
	H	65	15.472	18.984	-1.227
	H	66	12.113	22.900	-0.873
	H	67	12.753	22.915	-2.528
	H	68	15.059	22.342	-1.625
	H	69	7.889	20.256	3.207
	N	70	9.691	11.238	4.367
	N	71	11.155	12.754	3.660
	N	72	6.702	18.118	2.538
	N	73	7.445	20.365	4.119
	N	74	7.123	15.692	-2.787
	N	75	6.361	17.939	-0.229
	O	76	6.507	19.319	5.922
	O	77	5.834	23.263	5.509
	O	78	11.637	15.524	4.612
	O	79	4.937	16.386	-1.133
	O	80	4.606	19.025	2.788
	S	81	9.010	16.501	0.793
	S	82	9.787	17.975	3.809
	S	83	10.240	22.284	4.279
	S	84	12.560	20.656	-1.735
	S	85	16.037	18.518	1.090
	S	86	14.103	17.219	3.633
	S	87	12.400	17.828	0.360
	S	88	13.204	20.849	2.109
	S	89	9.801	20.388	0.851

References

1 B. Delley, *J. Chem. Phys.*, 1990, **92**, 508-517.

2 B. Delley, in *Modern density functional theory: a tool for chemistry*, eds. J. M. Seminario and P. Politzer, Elsevier, Amsterdam, 1995, p. 221-254

3 B. Delley, *J. Chem. Phys.*, 2000, **113**, 7756-7764.

4 I. G. Dance, in *Transition Metal Sulfur Chemistry: Biological and Industrial Significance*, eds. E. I. Stiefel and K. Matsumoto, American Chemical Society, Washington, DC, USA, 1996, p. 135-152

5 I. Dance, *J. Chem. Soc., Chem. Commun.*, 1998, 523-530.

6 I. Dance, *J. Am. Chem. Soc.*, 2005, **127**, 10925-10942.

7 I. Dance, *Dalton Trans.*, 2011, **40**, 6480-6489.

8 I. Dance, *Molecular Simulation*, 2008, **34**, 923-929.

9 I. Dance, *Molecular Simulation*, 2011, **37**, 257.

10 A. Volbeda, P. Amara, C. Darnault, J.-M. Mouesca, A. Parkin, M. M. Roessler, F. A. Armstrong and J. C. Fontecilla-Camps, *Proceedings of the National Academy of Sciences*, 2012, **109**, 5305-5310.

[11] S. Frielingsdorf, J. Fritsch, A. Schmidt, M. Hammer, J. Löwenstein, E. Siebert, V. Pelmenschikov, T. Jaenicke, J. Kalms, Y. Rippers, F. Lendzian, I. Zebger, C. Teutloff, M. Kaupp, R. Bittl, P. Hildebrandt, B. Friedrich, O. Lenz and P. Scheerer, *Nat Chem Biol*, 2014, **10**, 378-385.

[12] M.-E. Pandelia, D. Bykov, R. Izsak, P. Infossi, M.-T. Giudici-Orticoni, E. Bill, F. Neese and W. Lubitz, *Proceedings of the National Academy of Sciences*, 2013, **110**, 483-488.

[13] J.-M. Mouesca, J. C. Fontecilla-Camps and P. Amara, *Angewandte Chemie International Edition*, 2013, **52**, 2002-2006.

[14] M. Stein and S. Kaur-Ghumaan, *Biochemical Society Transactions*, 2013, **41**, 1317-1324.

[15] V. Pelmenschikov and M. Kaupp, *Journal of the American Chemical Society*, 2013, **135**, 11809-11823.
